# Optimal interventions in networks during a pandemic

**DOI:** 10.1007/s00148-022-00916-y

**Published:** 2022-08-13

**Authors:** Roland Pongou, Guy Tchuente, Jean-Baptiste Tondji

**Affiliations:** 1 Department of Economics, University of Ottawa, 120 University Private, Social Sciences Building, Ottawa, K1N 6N5 Ontario Canada; 2 School of Economics, University of Kent, Kennedy Building, Park Wood Road, Canterbury, CT2 7FS Kent UK; 3 NISER, 2 Dean Trench Street, Smith Square, London, SW1P 3HE UK; 4 Department of Economics, The University of Texas Rio Grande Valley, 1201 West University Drive, Edinburg, 78539 TX USA; 5 Center for African Studies, Harvard University, 1280 Massachusetts Avenue, Floor 3, Cambridge, MA 02138 USA

**Keywords:** COVID-19, Health-vs-wealth prioritization, Lockdown, Networks, Nursing homes, D85, E61, H12, I18, J15

## Abstract

We develop a model of optimal lockdown policy for a social planner who balances population health with short-term wealth accumulation. The unique solution depends on tolerable infection incidence and social network structure. We then use unique data on nursing home networks in the US to calibrate the model and quantify state-level preference for prioritizing health over wealth. We also empirically validate simulation results derived from comparative statics analyses. Our findings suggest that policies that tolerate more virus spread (laissez-faire) increase state GDP growth and COVID-19 deaths in nursing homes. The detrimental effects of laissez-faire policies are more potent for nursing homes that are more peripheral in networks, nursing homes in poorer counties, and nursing homes that operate on a for-profit basis. We also find that US states with Republican governors have a higher tolerable incidence level, but these policies tend to converge with a high death count.

**Supplementary Information:**

The online version contains supplementary material available at 10.1007/s00148-022-00916-y.

## Introduction

In this study, we develop a theory of optimal lockdown policy for a social planner who prioritizes population health over short-term wealth accumulation during a pandemic that spreads through networks of physical contacts. Using unique data on nursing home networks in the USA, we calibrate the model and quantify state-level preference for prioritizing health over wealth during the COVID-19 pandemic. We also uncover new results on the effects of network configuration, network centrality, and health policies on COVID-19 deaths in nursing homes.

The application of our model to the spread of COVID-19 is timely and fitting. COVID-19 has affected millions of individuals and claimed many lives globally. To reduce its spread, governments worldwide have relied massively on lockdown and social distancing policies (Buchholz 2020). While lockdown measures have had some positive results, the associated economic costs have been considerable (Marquez-Padilla and Saavedra 2022). The gross domestic product in both developed and developing countries has decreased significantly as a result of economic contraction (International Monetary Fund 2020). The significant costs associated with quasi-complete lockdowns have forced governments to think about alternative policies that are less costly, such as imposing quarantine measures only on certain individuals while letting others go back to work. The natural questions that arise are how do we design optimally targeted lockdown policies that account for social network structure, and how do these policies affect health and economic dynamics?

We address these questions for a society that, to a certain extent, prioritizes health over short-term wealth accumulation.^1^ The problem is formalized using an N-SIRD model with lockdown as follows. Agents (including individuals and social infrastructures) are connected through a weighted undirected network of physical contacts through which the virus is likely to spread. At any point in time, an agent is in one of the following health compartments: susceptible (S), infected (I), recovered (R), and dead (D). Susceptible agents can become infected, while infected agents can recover or die. To reduce the contagion, a social planner enforces a lockdown which modifies the structure of the prevailing social network. Susceptible, infected, and recovered agents can all be sent into lockdown at different individual probabilities. The disease dynamics follow an individual-based mean-field model for epidemic modeling on networks.^2^ The planner’s objective is to determine the lockdown policy that contains the spread of the disease below a tolerable infection incidence level, and that maximizes the present discounted value of real income (or alternatively, that minimizes the economic cost of the pandemic), in that order of priority. In other words, the social planner can allocate the “work-from-home” rights to achieve these goals.

An appeal of our approach to the social planner’s problem is that it does not force us to assign a precise monetary value to health or to life.^3^ Rather, it allows some flexibility in how to design policies, with clear health and economic goals in mind. For instance, the social planner could set an infection incidence level that allows to keep the number of infected individuals below hospitals’ maximum capacities. We apply our theoretical model to analyze: (1) the effect of network structure on the dynamics of optimal lockdown, infection, recovery, death, and economic costs; (2) the tradeoff between public health and wealth accumulation; and (3) how different measures of network centrality affect the probability of being sent to lockdown.

To solve the planner’s problem, we first characterize the disease dynamics in our epidemiological model and obtain a unique solution under classical conditions. The rates of infection, recovery, and death at any given time are functions of the lockdown variable and the initial network of contacts that captures social structure. The basic reproduction number, *R*_0_, plays a role in enforcing mitigation strategies, including lockdown, when facing a potential virus spread.^4^ The planner’s problem admits a unique solution that depends on both the infection incidence level tolerated by the planner and the prevailing network of physical contacts that characterizes the society. The tolerable infection incidence level and the network of physical contacts determine the disease dynamics and the economic costs of lockdown. The lockdown policies affect the total number of individuals experiencing infection during the outbreak.

Using simulations which rely on realistic early COVID-19 transmission rate data, we conduct several comparative static analyses of our theoretical findings. Our results show that a higher tolerable incidence level results in lower lockdown rates and a smaller loss in economic surplus. While this finding illustrates the health-vs-wealth tradeoff the social planner faces, it does not prescribe any resolution since the planning decision depends on the relative value assigned to population health and short-term economic conditions by society. We also illustrate how lattice, small-world, random, and scale-free network structures affect optimal lockdown probabilities and disease dynamics, respectively. Our simulation results show that the cumulative proportion of the population sent into lockdown is always higher in random and small-world network structures than in lattice and scale-free structures. These lockdown policies translate to different epidemic and economic cost dynamics for each network. We extend our analysis to examine the potential impact of *network density* (or the interconnections between agents in a network) in our N-SIRD model for a small-world network. Our simulations show that optimal lockdown probabilities increase with network density.^5^ Third, we illustrate how measures of network centrality affect optimal lockdown probabilities and disease dynamics. We present correlations between four network metrics—degree, eigenvector, betweenness, and closeness—and the average lockdown probabilities in a small-world network. Our simulation results suggest that individuals who are more central in such a network are more likely to be sent into lockdown. This implies that more restrictive lockdown policies have a greater effect on individuals who are more central in networks. Overall, our simulation results confirm the intuition that not *all* agents should be placed into full lockdown under the optimal policy (e.g., Gollier 2020a, 2020b, Acemoglu et al. 2021, Bosi et al. 2021, Chang et al. 2021, and ; Pestieau and Ponthière 2022).

We calibrate our model and test some of its key predictions using unique US nursing home networks data. The senior population in the USA accounts for a significant share of America’s COVID-19 deaths (National Center for Health Statistics 2020; Conlen et al. 2021). The surge of COVID-19 cases and deaths in nursing homes led the American federal government to ban nursing home visits on March 13, 2020. This restriction enabled researchers from the “Protect Nursing Homes” project to construct a US nursing homes network, using smartphone data (Chen et al. 2021). We use this network data in conjunction with other nursing home and US state-level datasets to calibrate the N-SIRD model.^6^ This calibration allows us to estimate the value of the tolerable COVID-19 infection incidence level (*λ*) for 26 US states. The parameter *λ* estimates the state government’s tolerable COVID-19 infection incidence, which by assumption represents the relative value the state assigns to population health and economic prosperity. As such, a higher value of *λ* describes a policy that tends more toward a “laissez-faire” regime (Gollier 2020a), indicating a planner’s inclination to maximize short-term economic gains. We find that the tolerable infection incidence level varies significantly across US states, making it possible to test some theoretical predictions of our model. We can attribute variations in *λ* to interstate heterogeneity and differences in states’ demographic and political characteristics, including the gender of a state’s governor, the party affiliation of a state’s governor, a state’s geographic location, and the number of COVID-19 fatalities in a state. These findings complement other studies showing an association between the political affiliation of a US state’s governor and COVID-19 cases and deaths (e.g., Neelon et al. 2021, and ; Baccini and Brodeur 2021).

Using regression-based analyses, we find that laissez-faire policies are associated with more COVID-19 deaths, consistent with the results from the simulation analysis. Nursing homes that are more central in the network experience more COVID-19 deaths. However, laissez-faire policies are more harmful to nursing homes that are more peripheral in the network. We also find that the detrimental effect of laissez-faire policies on COVID-19 fatalities in nursing homes is potent in poorer counties and in for-profit nursing homes. In another empirical test of the N-SIRD model with lockdown, we investigate the relationship between US states’ tolerable infection incidence level for COVID-19 and the state’s GDP growth for 2020. We find that laissez-faire policies are associated with higher GDP growth, consistent with our simulation results. Interestingly, we find that the positive economic effect of laissez-faire policies is reduced for US states with a Democratic governor.

Our paper contributes to several literatures. The epidemiological framework that we use to model the planning problem is a continuous-time individual-based mean-field model which belongs to the class of theoretical approaches for epidemic modeling on undirected heterogeneous networks; Pastor-Satorras et al. (2015) provide a review of these epidemiological models. This literature includes a class of mean-field models (Kephart and White 1992; Barabási et al. 1999; Green et al. 2006) and *N*-intertwined models via Markov theory in both discrete time (Ganesh et al. 2005; Wang et al. 2003) and continuous time (Van Mieghem et al. 2008). Asavathiratham (2001) and Garetto et al. (2003) review other general models for virus spread in networks based on Markov theory. These models extend the classical susceptible-infected-recovered (SIR) (Kermack and McKendrick 1927) and SIRD (Hethcote 2000) epidemic processes to heterogeneous networks. To this literature, we add a reversible lockdown state to model disease dynamics in an SIRD epidemic framework. The targeted lockdown policy allows the planner to achieve specific health and economic goals. In this perspective, our study contributes to the literature interacting epidemiology and economics to address a variety of issues.

Our model differs from previous approaches in two key respects: a lockdown variable and a weighted network of contacts that is not necessarily *random*.^7^ In this weighted network, we also assume that agents are heterogeneous with respect to the intensity of their connections and their individual characteristics. Most importantly, we introduce a *lexicographic approach* to the planning problem.

Our goal is to provide a dynamic economic and epidemiological model of lockdown, in which a planner must choose a lockdown policy which keeps infections below a certain threshold level at the minimum economic cost. Contrary to Bosi et al. (2021), who proposes a model where the planner imposes a single lockdown policy which remains constant over time, we propose a model where lockdown policy is dynamic, reversible, and subject to change over time. In this respect, our model is more in line with Gollier (2020a), Acemoglu et al. (2021), Alvarez et al. (2021), and Pestieau and Ponthière (2022).^8^

Our study is also connected to the economic literature on the design of optimal interventions in networks. Ballester et al. (2006) and Banerjee et al. (2013) examine the optimal targeting of key players (that is, the first individuals to receive a piece of information) in a network. Galeotti et al. (2020) analyze optimal interventions that change individuals’ private returns to investment in a network. Nganmeni et al. (2022) analyze stable, inclusive, and Pareto-efficient vaccine allocations in spatial networks. Our research question differs in that we study optimal lockdown interventions in a network. In our model, the choice of lockdown strictness operates to control the spread of infection through the network. By focusing on contagion, our paper relates to the studies of Young (2009) and Young (2011), who investigate the diffusion of innovations through networks. Our work is also connected to the models of social learning dynamics in Buechel et al. (2015) and Battiston and Stanca (2015), with the main difference being that infection diffusion is exogenous in these models. Our epidemiological model also complements and extends (Peng et al. 2020), by allowing for diffusion dynamics similar to Lloyd et al. (2006). Additionally, since our network structure is not necessarily random, we are able to develop new applications. Although we only apply our model to the COVID-19 pandemic, we believe that our theory has implications for other infections that spread through physical contacts. In line with Pongou and Serrano (2013), Chang et al. (2021), Fajgelbaum et al. (2021), Debnam Guzman et al. (2022), and Pongou et al. (2022), our study also contributes to the growing literature investigating the importance of network structure in the distributional effects of virus spread.

The remainder of this study is organized as follows. Section 2 presents the N-SIRD model with lockdown. Section 3 describes and solves the planning problem. Section 4 uses simulations to provide comparative statics analyses of our theoretical findings. Section 5 provides an empirical application of the theoretical model. Section 6 discusses some policy implications and offers concluding remarks. The Appendices contain complementary information for the N-SIRD model and additional simulation and empirical results.

## N-SIRD model with the lockdown

We describe the evolution of an epidemic that spreads through an undirected weighted and symmetric network of physical contacts, *A*. Time *t* is continuous, $t\in [0, \infty )$, and there is no vital dynamics so that a community of size *N* is constant through time: *N*(*t*) = *N* for all *t*.

### Social network structure

We represent *A* by the adjacency matrix (*A*_*i*,*j*_), where $A_{ij} = A_{ji} \in [0, \infty )$ represents the *weight* or *intensity* at which individuals *i* and *j* are connected in *A*, with *A*_*ij*_ = 0 if *i* = *j*. The intensity of connections is the primary source of heterogeneity between agents in the social network structure *A*.^9^ However, other characteristics may differentiate agents with the same number of connections. In Section 5, in which we apply our theory to US nursing home networks (Chen et al. 2021), a node is defined as a single nursing home. As such, nodes (nursing homes) have different surplus functions and can be either for-profit or not-for-profit.

### Health compartments

At any time *t*, individuals are divided into four compartments: susceptible *S*(*t*), infected *I*(*t*), recovered *R*(*t*), and deceased *D*(*t*), where *S*(*t*) + *I*(*t*) + *R*(*t*) + *D*(*t*) = *N*. For simplicity, we drop the time subscript of different compartments. Each individual *i* is in each of the four different compartments with the following probabilities: *s*_*i*_ = *P*(*i* ∈ *S*), *x*_*i*_ = *P*(*i* ∈ *I*), *r*_*i*_ = *P*(*i* ∈ *R*), and *d*_*i*_ = *P*(*i* ∈ *D*), with *s*_*i*_ + *x*_*i*_ + *r*_*i*_ + *d*_*i*_ = 1.

### Lockdown

We incorporate a lockdown variable to capture the fact that a social planner might decide to reduce the spread of the infection by enforcing a lockdown policy. This lockdown policy reduces the spread of infection by modifying the existing social network structure, *A*. Let *L* denote the lockdown state that is controlled by the social planner, and *l*_*i*_ = *P*(*i* ∈ *L*) denote the probability that a random individual *i* is sent into lockdown, with *l*_*i*_ = 1 designating full lockdown and *l*_*i*_ = 0 no lockdown. Intermediate values of *l*_*i*_ ∈ (0,1) represent less extreme cases.

### Virus spread

Susceptible individuals may become infected by coming into contact with infected individuals at a constant passing rate β. Individuals move from susceptible to infected, then either recover at rate *γ* or die at rate *κ*.^10^ We assume that a policy of full lockdown is 100% effective in curbing the contagion, i.e., full lockdown is similar to self-isolation.^11^ An individual in full lockdown is completely disconnected from all contacts. Thus, susceptible individuals in full lockdown in period *t* remain susceptible in the next period *t* + *𝜖*, *𝜖* positive and very small. Therefore, with lockdown, the probability of an individual *i* being infected is equal to the probability that they are susceptible (*s*_*i*_) and not sent into full lockdown (1 − *l*_*i*_ > 0) multiplied by the probability that a neighbor *j* is infected (*x*_*j*_ > 0) and is not sent into full lockdown (1 − *l*_*j*_ > 0), scaled by the connection intensity between *i* and *j* (*A*_*ij*_ > 0) and the contact rate β. It follows that the infinitesimal change in infection probabilities over time for individual *i* is:

$$\dot{x_{i}}= \upbeta s_{i}(1 -l_{i})\sum\limits_{j\in N} A_{ij} (1-l_{j})x_{j} - (\gamma +\kappa) x_{i}.$$

### Disease dynamics

The equation generated by $\dot {x_{i}}$ describes the law of motion of the infection probabilities for individual *i*. Any individual can be sent into lockdown regardless of whether the individual is susceptible, infected or recovered. For each *i* ∈ *N*, let *X*_*i*_ = (*x*_*i*_,*s*_*i*_,*r*_*i*_,*d*_*i*_)^*T*^ denote agent *i*’s health characteristics in the population, where *T* means “transpose.” We summarize the laws of motion of the variables of interest given the lockdown profile *l* = (*l*_*i*_)_*i*∈*N*_ by the following nonlinear system of ordinary differential equations:

$$\text{(ODE)}:\left\{ \begin{array}{l} \dot{s_{i}} = - \upbeta s_{i}(1 -l_{i})\sum\limits_{j\in N} [A_{ij} (1-l_{j}) x_{j}]\\ \dot{x_{i}} = \upbeta s_{i}(1 -l_{i})\sum\limits_{j\in N} [A_{ij} (1-l_{j})x_{j}] -(\gamma +\kappa) x_{i}\\ \dot{r_{i}} = \gamma x_{i}\\ \dot{d_{i}} = \kappa x_{i}\\ s_{i} + x_{i} + r_{i} + d_{i} =1 \end{array}\right.$$

where the initial value point (*x*_*i*_(0),*s*_*i*_(0),*r*_*i*_(0),*d*_*i*_(0)) is such that

$$x_{i}(0) \geq 0, \ s_{i}(0) \geq 0, \ r_{i}(0)\geq 0, \ d_{i}(0)\geq 0, \ \text{and} \ x_{i}(0) + s_{i}(0) + r_{i}(0)+ d_{i}(0)= 1.$$

We use the N-SIRD model with lockdown (ODE) to obtain qualitative insights into the transmission dynamics of the disease. Before using the model to simulate disease dynamics and evaluate control strategies in Sections 4 and 5, respectively, it is instructive to explore the model’s basic qualitative properties. First, we must establish that a solution for the system (ODE) exists. We demonstrate the existence of a solution for the system (ODE) in Proposition 1.

### **Proposition 1**

The system (ODE) admits a unique solution $\mathcal {S}^{*}= \mathcal {S}^{*}(l, A, \upbeta , \gamma , \kappa )$.

### *Proof*

See Online Appendix C.1.

Next, we carry out the analysis of the N-SIRD model in the feasible domain:

$${\Omega} = \{((x_{i})_{i\in N}, (s_{i})_{i\in N}, (r_{i})_{i\in N}, (d_{i})_{i\in N}) \in [0, 1]^{4n}: x_{i} + s_{i} + r_{i} + d_{i} \leq 1, 1 \leq i \leq n\}.$$

The domain Ω is positively invariant (i.e., solutions that start in Ω remain in Ω for all *t* ≥ 0). Hence, we can confirm that the system (ODE) is mathematically and epidemiologically well posed in Ω (Hethcote 2000).

### Equilibria and the basic reproduction number

To find equilibria in the system (ODE), we set each expression on the left-hand side of equations in (ODE) equal to zero. It follows that any equilibrium point constitutes a disease-free equilibrium point (DFE) in which the probability of infection is zero, i.e., *x*_*i*_ = 0 for all *i* ∈ *N*. For simplicity, we analyze the disease dynamics at the DFE *E*_0_ = (0,...,0,1,...,1,0,...0,...,0) in a completely susceptible population. One of the most fundamental concepts in epidemiology is the basic reproduction number, *R*_0_. The number, *R*_0_, describes the expected number of secondary cases produced by a typical infected individual during their entire period of infectiousness in a completely susceptible population. Following Diekmann et al. (1990) and Van den Driessche and Watmough (2002), only those in the infected compartments *I* are used in the calculation of *R*_0_. We use the next-generation matrix method to calculate *R*_0_. Formally, *R*_0_ is defined as the spectral radius of the next-generation matrix $\mathcal {A} {\mathscr{B}}^{-1}$, where $\mathcal {A}$ is the matrix of the rate of generation of new infections, and ${\mathscr{B}}$ is the matrix of transfer of individuals among the four health compartments. Following Van den Driessche and Watmough (2002), from the system (ODE), we write:

$$\dot{x_{i}} = \mathcal{A}_{i} - \mathcal{B}_{i}, \ \text{where}$$

$$\mathcal{A}_{i}= \upbeta (1-x_{i}-r_{i}-d_{i})(1 -l_{i})\sum\limits_{j\in N} [A_{ij} (1-l_{j})x_{j}], \ \text{and} \ \mathcal{B}_{i}= (\gamma +\kappa) x_{i}.$$

$\mathcal {A}$ is the Jacobian matrix, and it is given by $\mathcal {A}= [\frac {\partial \mathcal {A}_{i}} {\partial x_{j}} = \mathcal {A}_{ij}]_{E_{0}}$, and ${\mathscr{B}}= [\frac {\partial {\mathscr{B}}_{i}} {\partial x_{j}} = {\mathscr{B}}_{ij}]_{E_{0}}$, where *x* = (*x*_*j*_) = (*x*_1_,*x*_2_,...,*x*_*n*_). We have $\mathcal {A}_{ii} = -\upbeta (1-l_{i}) \sum \limits _{j\in N} [A_{ij} (1-l_{j})x_{j}]$ and $\mathcal {A}_{ij} = \upbeta A_{ij} (1-x_{i}-r_{i}-d_{i})(1 -l_{i}) (1-l_{j})$ for *j*≠*i*. At the equilibrium point *E*_0_, it holds that $\mathcal {A}_{ii}(E_{0}) =0$ and $\mathcal {A}_{ij}(E_{0}) = \upbeta A_{ij} (1 -l_{i}) (1-l_{j})$ for *j*≠*i*. Since *A*_*ii*_ = 0, we can write

$$\mathcal{A}_{ij} (E_{0}) = \upbeta A_{ij}(1 -l_{i}) (1-l_{j}), \ \text{for} \ 1\leq i, j \leq n.$$

It is straightforward to have ${\mathscr{B}}_{ij}(E_{0}) = (\gamma + \kappa ) \delta ^{ij}$, where *δ*^*ij*^ = 1 if *i* = *j*, and *δ*^*ij*^ = 0 otherwise. It follows that ${\mathscr{B}}_{ii}= \gamma + \kappa$ and ${\mathscr{B}}_{ii}^{-1}= \frac {1}{\gamma + \kappa }$, for all 1 ≤ *i* ≤ *n* such that

$$\mathcal{B}= diag (\mathcal{B}_{11},..., \mathcal{B}_{ii},..., \mathcal{B}_{nn}) \ \text{and} \ \mathcal{B}^{-1}= diag (\mathcal{B}_{11}^{-1},..., \mathcal{B}_{ii}^{-1},..., \mathcal{B}_{nn}^{-1}).$$

Therefore, $\mathcal {A}{\mathscr{B}}^{-1} \equiv {\mathscr{M}} = [{\mathscr{M}}_{ij}]_{1\leq i, j \leq n}$, where ${\mathscr{M}}_{ij}= \frac {\upbeta }{\gamma + \kappa } A_{ij} (1-l_{i})(1-l_{j})$, and $R_{0} = \rho ({\mathscr{M}}):= {\max \limits } \{|e|: e \ \text {is an eigenvalue of} \ {\mathscr{M}} \}$. In a fully homogeneous connected society (e.g., a lattice network), it holds that *A*_*ij*_ = 1 for all agents *i* and *j* (*i*≠*j*), and without any non-pharmaceutical intervention such as lockdown, $R_{0}= \frac {\upbeta }{\gamma + \kappa } (n-1)$. Since *A* is undirected, it holds that *A*_*ij*_ = *A*_*ji*_, so that ${\mathscr{M}}_{ij}= {\mathscr{M}}_{ji}$ for all *i* and *j*. Additionally, since all the values *A*_*ij*_, 1 − *l*_*i*_, and 1 − *l*_*j*_ are real and non-negative, it follows that ${\mathscr{M}}$ is a non-negative symmetric real matrix. Therefore, all of its eigenvalues and eigenvectors are real. Since the diagonal of ${\mathscr{M}}$ consists of zero, it holds that the trace of ${\mathscr{M}}$ is zero (recall that the trace of ${\mathscr{M}}$ is the sum of its eigenvalues). Given that the determinant of ${\mathscr{M}}$, which is the product of its eigenvalues, is not necessarily zero, it follows that *R*_0_ is positive. The following result provides the asymptotic stability analysis of continuum of the disease-free equilibrium *E*_0_.

### **Proposition 2**

The continuum of *DFE* *E*_0_ of the system (ODE) is locally asymptotically stable if *R*_0_ < 1, but unstable if *R*_0_ > 1.

### *Proof*

See Online Appendix C.2.

The epidemiological interpretation of Proposition 2 is that a small invasion of virus-infected agents will not generate an epidemic outbreak in society when the basic reproduction number is below 1. However, when *R*_0_ > 1, the epidemic rises to a peak and then eventually declines to zero. Proposition 2 also suggests that a social planner may need lockdown policies to reduce contagion only when *R*_0_ is expected to be greater than 1. For instance, when *R*_0_ = 2, one infected agent will, on average, infect two different agents during their period of infectiousness. Following this sequence, we expect each new infected agent to transmit the virus to two other susceptible agents. Therefore, without any intervention and mitigation measures, the contagion may spread exponentially and cause significant health and economic costs. This explains why lockdown and quarantine policies, together with other non-pharmaceutical interventions such as physical distancing, mask wearing, and hygiene measures, are the immediate solutions that policymakers turn to at the onset of any pandemic when pharmaceutical treatments are not available. An additional finding in Online Appendix A illustrates the pivotal role of *R*_0_ and the next-generation matrix ${\mathscr{M}}$ in determining the final size of the epidemic in the N-SIRD model with the lockdown. In response to a larger size of *R*_0_, enforcing a lockdown state to reduce physical contacts between targeted individuals with other agents in the population changes the disease dynamics in the social network structure. As we will show throughout, such a non-pharmaceutical decision could help planners fight the virus spread at a minimum cost by allowing some agents to continue supplying services in the economy.

## The planning problem: optimal lockdown

The unique solution to the nonlinear system (ODE) presented in Section 2 depends on the network structure, *A*, and the lockdown variable, *l*. The planning problem consists of choosing the optimal lockdown policy *l* such that infections are kept below the chosen threshold value at the lowest economic cost possible. Importantly, the planner always *prioritizes* keeping infections under the infection incidence threshold. This means that they are willing to pay an infinitely high economic price to keep infections below their threshold level. Formally, the planner’s problem consists of choosing *l* that:

1. Contains the infection *incidence* level (or the relative number of new infections) below a *tolerable* threshold *λ*; and
2. Minimizes the economic costs of the lockdown policy, in that order of priority.

Below, we formalize this lexicographic objective problem.

### Containing the spread of infection

Using $\dot {x_{i}}$ in the system (ODE), the first objective of the planner is to select a lockdown policy *l* such that:

1 $$\dot{x_{i}}\equiv \dot{x_{i}}(l) \leq \lambda, \ \text{where} \ \lambda \ \text{is a non-negative parameter}.$$

Note that the system (ODE) together with Eq. 1 admits at least one solution. Consider the policy *l* where each individual is sent into full lockdown, i.e., *l*_*i*_(*t*) = 1 for all *i* ∈ *N* and *t*. Then, $\dot {x_{i}}(l)=-(\gamma +\kappa ) x_{i}$. Therefore, given any *λ* ≥ 0, it follows that $\dot {x_{i}}(l) \leq \lambda$. However, this extreme solution induces significant social and economic costs. In practice, the upper bound of the parameter *λ* could be equal to the basic reproduction number without any lockdown policy, ${R_{0}^{v}} = \rho ({\mathscr{M}}^{v})$, where ${\mathscr{M}}^{v} = [\frac {\upbeta }{\gamma + \kappa } A_{ij}]_{1\leq i, j \leq n}$. Given that lockdown implies a reduction of economic activities, an economically focused planner might tolerate a value of *λ* close to ${R_{0}^{v}}$. In contrast, a cautious (or prudent) planner who prioritizes health over economic prosperity may only tolerate infection incidences *λ* that fall behind the basic reproduction number *R*_0_.

### Minimizing the economic costs of lockdown

The planner’s second-order objective is to minimize the economic costs of lockdown by choosing from the set of policies that satisfy the first objective, the policy that maximizes the present discounted value of aggregate wealth or surplus. To assess the economic effects of lockdown in the population during a pandemic, we consider a simple production economy that we describe as follows.

### Inputs

At any given period *t*, each individual *i* possesses a capital level *k*_*i*_, and a labor supply *h*_*i*_. We assume, as in most SIR models, that individuals who recover from the infection are immune to the virus and must be released to the workforce. It follows that individuals in compartments *S*, *I*, and *R* are the only potential workers in the economy. The individual labor supply depends on individuals’ health compartments and their probability of being in lockdown: *h*_*i*_ = *h*_*i*_(*s*_*i*_,*x*_*i*_,*r*_*i*_,*d*_*i*_,*l*_*i*_), with *h*_*i*_ assumed to be continuous and differentiable in each of its input variables. We assume that *h*_*i*_ is non-decreasing in the probabilities of being susceptible and recovered: $\frac {\partial h_{i}}{\partial s_{i}}\geq 0$ and $\frac {\partial h_{i}}{\partial r_{i}}\geq 0$. In contrast, labor supply is non-increasing in the probabilities of being infected and deceased and is also non-increasing in lockdown strictness: $\frac {\partial h_{i}}{\partial x_{i}}\leq 0$, $\frac {\partial h_{i}}{\partial d_{i}}\leq 0$, and $\frac {\partial h_{i}}{\partial l_{i}}\leq 0$. Naturally, an individual who is working despite being infected produces less compared to an otherwise identical individual who is healthy. Without loss of generality, we assume that capital is constant over time (*k*_*i*_(*t*) = *k*_*i*_, for each *t*), and labor is a variable input in the production function.

### Output

Capital combines with labor to generate output, *y*_*i*_, based on a production function: *y*_*i*_ = *y*_*i*_(*k*_*i*_,*h*_*i*_) = *y*_*i*_(*k*_*i*_,*s*_*i*_,*x*_*i*_,*r*_*i*_,*d*_*i*_,*l*_*i*_). We assume that *y*_*i*_ is continuous and differentiable in each of its input variables. Moreover, we make the following natural assumptions: $\frac {\partial y_{i}}{\partial k_{i}} \geq 0$, $\frac {\partial y_{i}}{\partial s_{i}} \geq 0$, $\frac {\partial y_{i}}{\partial x_{i}} \leq 0$, $\frac {\partial y_{i}}{\partial r_{i}} \geq 0$, $\frac {\partial y_{i}}{\partial d_{i}} \leq 0$, $\frac {\partial y_{i}}{\partial l_{i}} \leq 0$, and $\frac {\partial {y_{i}^{2}}}{\partial ^{2} v}\leq 0$, for each *v* ∈{*k*_*i*_,*s*_*i*_,*x*_*i*_,*r*_*i*_,*d*_*i*_,*l*_*i*_}. Other important variables in the problem include the individual cost of one unit of labor (*w*_*i*_), the price per unit of output (*p*_*i*_), and the social planner’s discount rate (*δ*).

### Aggregate surplus

With the above information, agent *i*’s surplus function, *W*_*i*_, is given as *W*_*i*_(*k*_*i*_,*s*_*i*_,*x*_*i*_,*r*_*i*_,*d*_*i*_,*l*_*i*_) = *p*_*i*_*y*_*i*_(*k*_*i*_,*s*_*i*_,*x*_*i*_,*r*_*i*_,*d*_*i*_,*l*_*i*_) − *w*_*i*_*h*_*i*_(*s*_*i*_,*x*_*i*_,*r*_*i*_,*d*_*i*_,*l*_*i*_). The planner chooses the lockdown profile *l* = (*l*_*i*_)_*i*∈*N*_ ∈ [0,1]^*n*^ to maximize the present discounted value of aggregate surplus:

$$\begin{array}{lllll} W(k, s, x, r, d, l): & = \int \limits_{0}^{\infty} e^{-\delta t} \left\{\sum \limits_{i \in N} W_{i}(k_{i}, s_{i}, x_{i}, r_{i}, d_{i}, l_{i}) \right\} dt\\ & = \sum \limits_{i \in N} \left\{ \int \limits_{0}^{\infty} e^{-\delta t}\left(p_{i} y_{i}(k_{i}, s_{i}, x_{i}, r_{i}, d_{i}, l_{i}) - w_{i} h_{i}(s_{i}, x_{i}, r_{i}, d_{i}, l_{i})\right) dt\right\}. \end{array}$$

### The social planner’s problem

We recall that *X*_*i*_ = (*x*_*i*_,*s*_*i*_,*r*_*i*_,*d*_*i*_)^*T*^ represents agent *i*’s health characteristics in the population. Given a tolerable infection incidence *λ*, the planner’s task is to choose the optimal admissible lockdown path $l_{i}^{*}(t)$, for each agent *i* ∈ *N*, in period *t*, which along with the associated optimal admissible state path $X_{i}^{*}(t)$ will maximize the objective functional *W*. Using optimal control theory, we can formalize the social planner’s problem as:

2 $$\begin{aligned} & \underset{(l_{i})_{i\in N}}{\text{Maximize}} & & \int \limits_{0}^{\infty} e^{-\delta t} \sum \limits_{i\in N} \left\{ p_{i} y_{i}(k_{i}, s_{i}, x_{i}, r_{i}, d_{i}, l_{i}) - w_{i} h_{i}(s_{i}, x_{i}, r_{i}, d_{i}, l_{i})\right\} dt \\ & \text{subject to} & & \text{(ODE) and} \ \dot{x_{i}} \leq \lambda, \ i\in N \\ & \text{and} & & \ l_{i}(t) \in [0, 1] \ \text{for all} \ i \in N \ \text{and} \ t. \end{aligned}$$

We have the following result.

### **Proposition 3**

The social planner’s problem (2) has a unique solution.

### *Proof*

See Online Appendix C.3.

Proposition 3 states the existence and uniqueness of a solution to the social planner’s problem. In Online Appendix B, we extend the analysis of problem (2) that proves useful in showing how we obtain our simulated results. Note that determining a closed-form solution to the planning problem (2) is intractable. This is justified by the complex and stochastic nature of the system (ODE) that characterizes our N-SIRD model. To gain some insight into the optimal lockdown policy and the resulting disease and costs dynamics , we follow Alvarez et al. (2021), Acemoglu et al. (2021), and Gollier (2020a), and resort to simulations in Section 4. First, in Section 4.1, we vary the tolerable infection incidence *λ* to illustrate the tradeoff between health and wealth. Unlike Bosi et al. (2021) who proposes a constant optimal lockdown policy to curve the contagion, our lockdown policy is dynamic, and more in line with Alvarez et al. (2021), Acemoglu et al. (2021), and Gollier (2020a). We differ from Alvarez et al. (2021) and Acemoglu et al. (2021) by not constraining the lockdown probability by an upper bound less than one, which situates our study more in line with Bosi et al. (2021).^12^ In our model, a planner could lock down all of society if they found it optimal to do so. However, this case corresponds to a purely epidemiological model and our findings illustrate that full lockdown is not an optimal solution. Second, in Section 4.2, we illustrate how network configuration affects the disease dynamics and their impact on the economy, by changing the nature of the network structure *A*. Similarly, we also illustrate in Section 4.3 the effects of network centrality on individual lockdown probabilities.

## Comparative statics: a simulation-based analysis

We choose the parameters in the N-SIRD model to match the dynamics of the infection and early studies on the COVID-19 pandemic and the period in which the researchers at the Protect Nursing Homes gathered the data to build the US nursing home networks. Following Alvarez et al. (2021), we use data from the World Health Organization (WHO) made public through the Johns Hopkins University Center for Systems Science and Engineering (JHU CCSE). The contact rate β is assumed to be 0.2. The lifetime duration of the virus is assumed to be 18 days (e.g., Acemoglu et al. 2021 and the references therein). Given the information from JHU CCSE access on May 5, 2020, the proportion of recovered closed cases was around 70% for the USA, 93% for Germany, and 86% for Spain. Thus, we assume that the parameter governing the recovery of an infected patient is given by $\gamma = \frac {0.8}{18}$, and the parameter governing the death dynamics is given by $\kappa = \frac {0.2}{18}$.

### Calibrating the production function

We consider the following functional forms for the labor function (*h*) and the production function (*y*):

3 $$\begin{array}{@{}rcl@{}} h_{i}(s_{i}, x_{i}, r_{i}, d_{i}, l_{i}) & =& (1 + \phi_{i} s_{i} r_{i} (1-x_{i})(1-d_{i})) (1- \varphi_{i} l_{i}), \end{array}$$

4 $$\begin{array}{@{}rcl@{}} y_{i}(k_{i}, s_{i}, x_{i}, r_{i}, d_{i}, l_{i}) & =& k_{i}^{\alpha_{i}}h_{i}^{1-\alpha_{i}}, \end{array}$$

where *ϕ*_*i*_ ∈ [0,1] determines the direct effect on the rate of change in the labor supply when individual *i* is in one of the natural health compartments, *S*, *I*, *R*, and *D*. The parameter *φ*_*i*_ ∈ [0,1] represents the direct effect on the labor supply which occurs when individual *i* is placed in lockdown, with this effect assumed to be non-positive. When *d*_*i*_ = *P*(*i* ∈ *D*) = 1, we should have *l*_*i*_ = 0 so that *h*_*i*_(*s*_*i*_,*x*_*i*_,*r*_*i*_,1,0) = 0. In Eq. 4, *α*_*i*_ is the elasticity of output with respect to the capital, and 1 − *α*_*i*_ is the elasticity of output with respect to labor. The functions *h*_*i*_ in Eq. 3 and *y*_*i*_ in Eq. 4 satisfy the standard conditions mentioned in Section 3.

Our choice of the Cobb-Douglas function as a parametric estimate of the production function is motivated by our empirical analyses in Section 5. Our consideration is also more in line with several studies that argue that the Cobb-Douglas function is a standard parameterization of production functions in the literature (Douglas 1976), especially in the context of primary care (Wichmann and Wichmann 2020), and nursing homes (Reyes-Santías et al. 2020). Using the recent data collected by Chen et al. (2021) on US nursing homes, we approximate a typical nursing home’s production function as $y_{i} = k_{i}^{\alpha _{i}} h_{i}^{1-\alpha _{i}}$, where *y*_*i*_ is the total number of residents (proxies the nursing home’s output) who receive care, *k*_*i*_ is the total number of beds (proxies the capital), and *h*_*i*_ is the number of occupied beds (proxies the labor supply). In our simulations, we consider $\alpha _{i}= \frac {1}{3}$. For more details on our estimation approach of a nursing home’s production function, we refer to Online Appendix F.1.

In all simulations, we consider *ϕ*_*i*_ = 0 and *φ*_*i*_ = 1, so that *h*_*i*_(*s*_*i*_,*x*_*i*_,*r*_*i*_,*d*_*i*_,*l*_*i*_) ≈ (1 − *l*_*i*_) and we have a stationary working population. In the context of nursing and long-term care homes, we can justify the labor supply’s approximation, *h*_*i*_ = 1 − *l*_*i*_. The connection between two nursing homes is determined by at least one signal received from a smartphone in both houses. Given the regulatory and staffing structures of US nursing homes, most workers in nursing homes would not be able to work remotely. In the context of our model, this implies that the labor supply will equal zero if a nursing home is in full lockdown, i.e., *h*_*i*_ = 0 when *l*_*i*_ = 1. The choice *h*_*i*_ = 1 − *l*_*i*_ is therefore an appropriate choice for the labor supply function, since it satisfies all the standard conditions mentioned above and allows for a smooth computational time process in our simulation analyses. Regarding the surplus function, we assume that $w_{i}=\frac {1}{3} p_{i}$, for each agent *i*, and assume that the level of capital is the same for all agents at all time periods. This level of capital is normalized to *k*_*i*_ = 1. The annual interest rate is assumed to be equal to 5%. In our simulation analyses, we assume for simplicity that networks are represented by binary adjacent matrices *A* = (*A*_*ij*_), where *A*_*ij*_ = 1 if agents *i* and *j* are connected, and *A*_*ij*_ = 0, otherwise.^13^ In Online Appendix D, we offer additional explanations of the simulations that we use to conduct our comparative statics analyses.

### Infection incidence control and optimal lockdown policy—the health-vs-wealth tradeoff

**Fig. 1:**
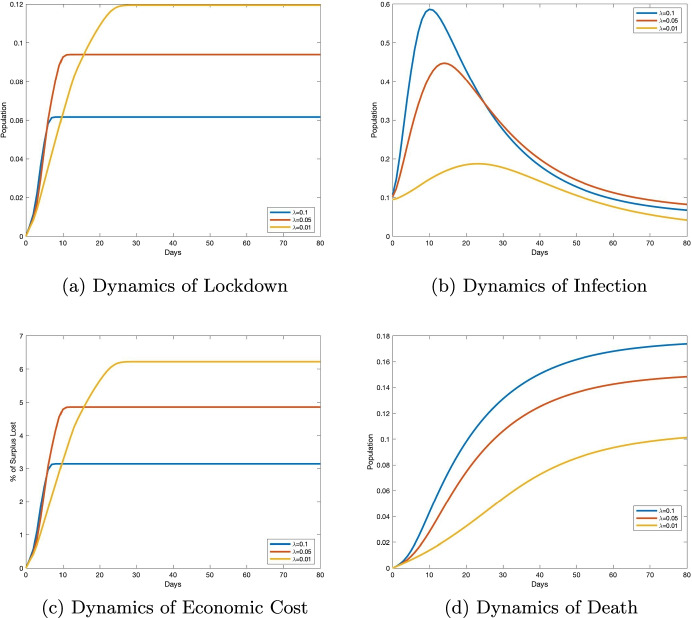
Health versus wealth tradeoff in a small-world network. Notes: We perform three sets of simulations with three different values of the tolerable infection incidence *λ*: 0.01, 0.05, and 0.1. The results are displayed in a two-dimensional graphic, with days on the horizontal axis, and the percentage of population affected for the variable (infection, lockdown, or death) on the vertical axis. In each period, a point in the graphic represents the average value of individual probabilities. For the economic cost, the vertical axis represents the percentage of economic surplus lost relative to the economy without the pandemic. Each graph shows three curves corresponding to three dynamics for a single variable of interest and a given value of *λ*. All variability within each curve in each graph is a result of the stochastic nature of transmission and not of variation in the network or in *λ*

In our first comparative statics analysis, we illustrate the effect of changing the tolerable infection incidence level on the optimal lockdown policy and describe the tradeoff between maintaining the desired level of population health and minimizing short-term economic costs. We consider an economy of *n* = 1000 agents connected through a *small-world network* (Watts and Strogatz 1998) with 2 × *n* edges (*A* is fixed). In the planning problem, we vary the tolerable infection incidence, *λ*, between 0.01, 0.05, and 0.1. Figure 1 presents the simulation results for this exercise.

#### Simulation results

Figure 1a illustrates that the optimal cumulative lockdown rate increases with a lower infection incidence level. This rate was around 6% for an incidence level of 0.1, 9% for an incidence level of 0.05, and 12% for an incidence level of 0.01. What emerges from these numbers is that the relationship between the tolerable incidence level and the cumulative lockdown rate is not linear. As the tolerable infection incidence level decreases, the fraction of the population sent into lockdown increases, with the absolute value of this increase being smaller than the absolute value of the decrease. The optimal lockdown policy resulting from a given tolerable infection incidence level translates into corresponding dynamics for infection, death, and economic cost. In particular, Fig. 1b shows that a lower tolerable incidence level results in lower infection and death rates (see Fig. 1b and d). Figure 1c illustrates the tradeoff between population health and economic well-being. A lower tolerable infection incidence level increases the economic cost of the pandemic. Indeed, if the tolerable infection incidence level is low, more individuals must be sent into lockdown, which decreases individuals’ productiveness in the economy; this in turn produces a significant loss in terms of economic surplus. For instance, when the tolerable incidence decreases from 0.1 to 0.05, the fraction of the economic surplus lost to the pandemic increases from around 3 to over 5%; and a further decrease of the tolerable incidence level to 0.01 induces an economic surplus loss of around 6 percent. It follows that maintaining a lower infection incidence threshold is achieved at the expense of short-term economic prosperity.

#### Robustness

In Online Appendix G, we replicate the simulation results in Fig. 1 for scale-free, random, and lattice networks, in Figs. G1, G2, and G3, respectively. We also replicate Fig. 1 using recent epidemiological data on the COVID-19 Delta variant (see Fig. G4 in Online Appendix G). We find that all these additional simulation results are qualitatively consistent with the lockdown, disease, and economic costs dynamics described in Fig. 1.

### The role of network configuration

**Fig. 2:**
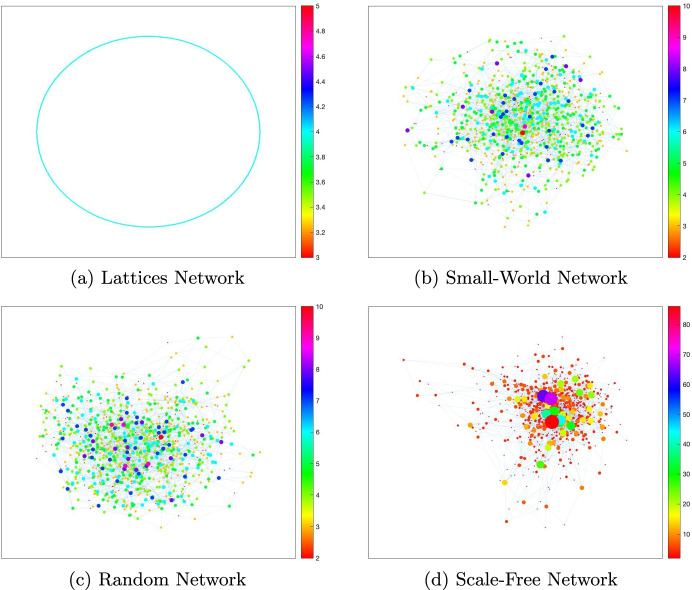
Simple network structures. Notes: Four distinct network types containing 1000 agents. Random networks display homogeneity of agent-level network properties and low clustering. Lattices are homogeneous at the agent level, and they show high clustering. Lattice networks also exhibit long path lengths, i.e., it takes many steps to move between two randomly selected agents, whereas random networks have short path lengths. Small-world networks display high clustering and short path lengths. Scale-free networks capture different levels of heterogeneity (for example, super-spreaders) in populations. In all four graphs, the average number of contacts per agent is 2. In each network, we represent agents with high contacts by larger dots, and we shade each node according to its number of direct contacts using the scale beside each graph

In Section 4.2, we fix the tolerable infection incidence *λ* to 0.01, and we vary the structure of network configuration, *A*, in the planning problem. For the sake of concreteness, we contrast four *idealized* network configurations (Keeling and Eames 2005), namely a *lattice network* (Fig. 2a), a *small-world network* (Fig. 2b), a *random network* (Fig. 2c), and a *scale-free network* (Fig. 2d). These network types are some of the most frequently used to model disease transmission (see, e.g., Keeling and Eames 2005 and the references therein for a review of these networks). According to Keeling and Eames, “Each of these idealized networks can be defined in terms of how individuals are distributed in space (which may be geographical or social) and how connections are formed, thereby simplifying and making explicit the many and complex processes involved in network formation within real populations” (Keeling and Eames 2005, p. 299). Following this viewpoint, the networks in Fig. 2 represent four societies, each of which contains 1000 agents. These societies are assumed to be identical in all ways except the configuration of their contact network. The four network configurations differ in their clustering of connections and their path lengths between nodes, two essential factors in disease spread.

#### Simulation results

**Fig. 3:**
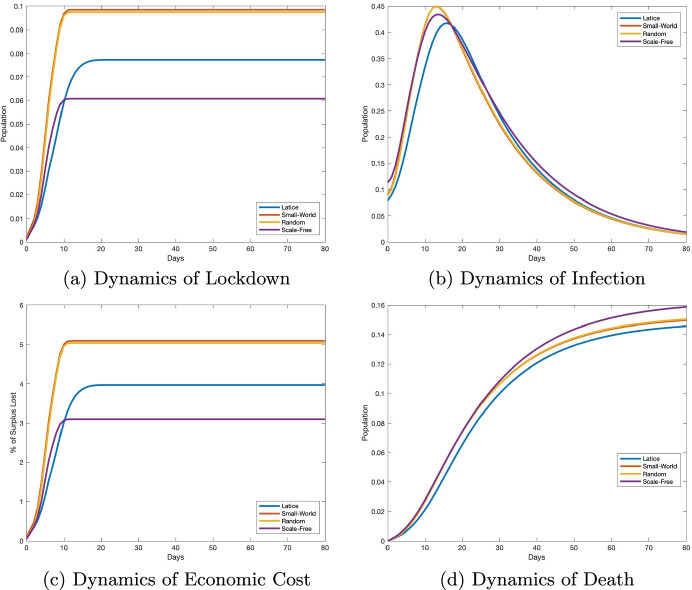
Optimal disease and economic cost dynamics in networks. Notes: N-SIRD epidemic process, lockdown, and economic cost dynamics on the four network types shown in Fig. 2. Each graph shows four curves corresponding to four networks for a single variable of interest. All variability within each curve in each graph is a result of the stochastic nature of transmission and not variation in the network. In the simulation, we assume that the tolerable infection incidence *λ* = 0.01. The results are displayed in a two-dimensional graphic, with days in the horizontal axis, and the percentage of population affected for the variable (infection, lockdown, or death) illustrated on the vertical axis. In each day, a point in the graphic represents the average value of individual probabilities. For the economic cost, the vertical axis represents the percentage of economic (or surplus) loss relative to the economy without the pandemic. Based on the simulation results (Fig. G4 in Online Appendix G) that we obtain by replicating Fig. 1 with the COVID-19 Delta variant in the small-world network, we conjecture that a replication of Fig. 3 would yield qualitatively consistent results

We represent the simulation results in these idealized networks in Fig. 3. From Fig. 3, we observe that both the epidemic dynamics and the economic costs of the disease are similar in the random network and small-world network structures. We can explain this similarity by the fact that short path lengths characterize both small-world and random networks. We illustrate the respective optimal lockdown policies in Fig. 3a for these four societies. The cumulative proportion of the population sent into lockdown peaks and flattens much earlier in the scale-free network society than in the lattice and small-world networks. At the onset of the pandemic, the lockdown is slightly stricter in the scale-free network compared to the lattice network. However, lockdown is always higher in random and small-world network configurations compared to lattice and scale-free configurations. The lockdown dynamics described in Fig. 3a respond to the disease dynamics that we illustrate in Fig. 3b for infection, and Fig. 3d for death. We observe that the reduction in initial growth in infection is more substantial in lattice networks compared with other networks. This is because a high spatial clustering of connections drives a more rapid saturation of local environment (Keeling and Eames 2005). In addition, findings from theoretical models of disease spread through scale-free-networks indicate that infection is generally concentrated among agents with the highest number of connections (Pastor-Satorras and Vespignani 2001; Newman 2002; Chang et al. 2021). Therefore, sending these potential super-spreaders into lockdown can significantly reduce the spread of contagion. Our optimal lockdown policy is consistent with these findings since our simulation results suggest that placing highly connected hubs or agents in lockdown can significantly reduce spread in a scale-free network. Once they are in lockdown, the speed of infection from one individual to another is reduced (a simple example is a situation in which agents are connected through a star network). The situation is different in the small-world and random network societies, where short path lengths suggest a rapid spatial spread of disease. In these network structures, containing the contagion below a chosen infection incidence level requires more severe lockdown measures than in the scale-free network. As the epidemic continues, the dynamics of surplus loss that we represent in Fig. 3c, due to the pandemic, are also different across the four networks, with random and small-world networks suffering the most severe economic costs, as a result of severe lockdowns. The lowest lockdown in scale-free network (Fig. 3a) results in more infection and deaths in the long run (Fig. 3d).^14^

#### Robustness with network density

**Fig. 4:**
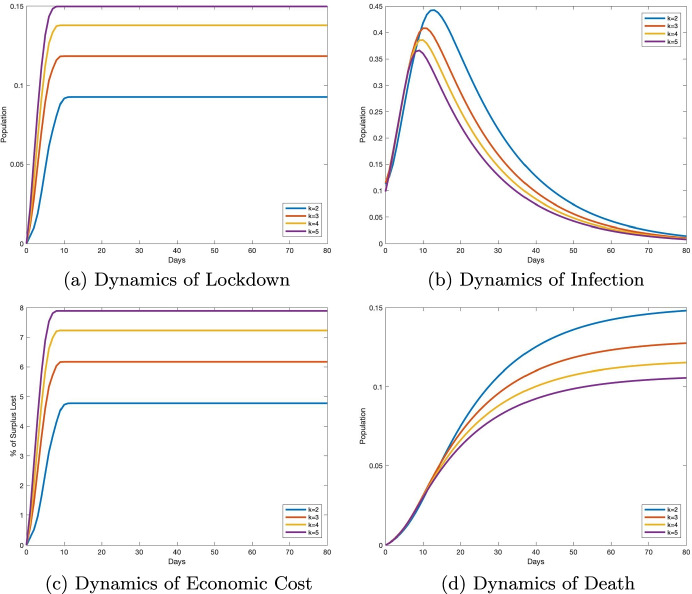
Optimal disease and cost dynamics in a small-world network with different densities. Notes: In our simulations, we assume that *λ* = 0.01. The results are displayed in a two-dimensional graphic, with days in the horizontal axis, and the percentage of population affected for the variable (infection, lockdown, or death) illustrated on the vertical axis. In each period, a point in the graphic represents the average value of individual probabilities. For the economic cost, the vertical axis represents the percentage of economic surplus lost relative to the economy without the pandemic. The density of network *A*_*k*_ is $d(A_{k})= \frac {2k}{n-1}$, where the parameter *k* represents the average number of connections per agent in network *A*_*k*_, and *n* number of nodes. Based on the simulation results (Fig. G4 in Online Appendix G) that we obtain by replicating Fig. 1 with the COVID-19 Delta variant in the small-world network, we conjecture that a replication of Fig. 4 would yield qualitatively consistent results

Following the comparative statics analyses on network topology described in Fig. 3, one might be interested in knowing how *network density* could affect the optimal lockdown policy, and therefore, the disease dynamics. To answer this question, we consider a society, *A*_*k*_, consisting of *n* = 1000 agents connected through a small-world network (Watts and Strogatz 1998) with *k* × *n* edges, where *k* represents the average number of connections per agent in the society. The density *d*(*A*_*k*_) of the network *A*_*k*_ measures how many ties between agents exist compared to how many ties between agents are possible, given the number of nodes, *n*, and the number of edges, *k* × *n*. Since *A*_*k*_ is an undirected network, $d(A_{k})= \frac {2k}{n-1}$, and the network becomes more *dense* as *k* increases (i.e., there is an increase in the number of connections between agents). Figure 4 represents the simulation results in society *A*_*k*_, when *k* ∈{2,3,4,5}. The optimal lockdown dynamics displayed in Fig. 4a indicate that lockdown probabilities increase with network density. The social planner justifies this increase in lockdown probability by the fact that the infection rate is, as portrayed in Fig. 4b, higher in more dense societies at the onset of the pandemic. As the pandemic evolves, strict lockdown is effective in containing the infection so that, in the long run, less dense societies bear a higher number of deaths relative to more dense societies in Fig. 4c. Similarly to Fig. 3, stricter lockdowns result in fewer economic transactions and, as a result, more dense networks suffer a more significant loss in economic surplus. This phenomenon is displayed in Fig. 4c.

#### Implications

Our simple experiment in Section 4.2 highlights the fact that network configuration should be a key factor in designing optimal lockdown policies during a pandemic like COVID-19. These non-pharmaceutical policies have implications for both health dynamics and economic costs. Indeed, our illustrations are consistent with other studies showing that network configuration plays an essential role in the spread of infection and diffusion of information (e.g., Keeling and Eames 2005, Pongou and Serrano 2013, 2016, and recently, Kuchler et al. 2021, and ; Chang et al. 2021). The numerical analysis also suggests that the wide range of variation in COVID-19 outcomes observed across countries, and across communities within countries, could be explained by differences in their network configuration. Several studies analyze the differences in COVID-19 outcomes between countries worldwide and communities within countries or regions. For comparisons among countries, see, e.g., Balmford et al. (2020) and Sorci et al. (2020); and for cross-community comparisons in COVID-19 outcomes in the USA, see, e.g., Chang et al. (2021) and Hong et al. (2021).

### Network centrality and optimally targeted lockdown

Our third comparative statics analysis highlights how lockdown policies can be optimally targeted at individuals based on their characteristics. The specific individual characteristic we consider is an individual’s centrality in their contact network. In general, certain agents occupy more central positions than others in the prevailing contact network of a networked economy (see, e.g., Chang et al. 2021). This can be due to a variety of factors, including the distinct social and economic characteristics of each individual. It is argued that individuals who occupy more central positions in networks are more likely to be infected and to spread an infection, e.g., Anderson and May (1992), Pastor-Satorras and Vespignani (2001), Newman (2002), Hethcote and Yorke (2014), Pongou and Tondji (2018), and Rodrigues (2019). This suggests that an optimal lockdown policy should be targeted at more central agents in a network. However, various measures of network centrality exist, and it is not clear which of these measures is most predictive in the context of a pandemic like COVID-19.

To address this issue, we consider four popular network metrics: degree centrality, eigenvector centrality, betweenness centrality, and closeness centrality. For clarity, we will briefly define each of these four measures of network centrality in Online Appendix E. To answer how each of the aforementioned network metrics predicts the probability of lockdown, we consider a society in which agents are connected through a small-world network with 2 × *n* edges. Agents occupy distinct positions in this network, resulting in some agents being more central than others. For robustness, our simulation analysis assumes three different values for the tolerable infection incidence *λ*: 0.01, 0.05, and 0.1.

#### Simulation results

Table 1 reports the correlation between each of our network metrics and the average optimal lockdown probabilities for different values of the tolerable infection incidence, *λ*, in a small-world network. Our simulation results in Table 1 suggest that the four centrality measures positively correlate to the likelihood of lockdown under the optimal lockdown policy. This correlation is statistically significant, as implied by the different *p*-value statistics. Moreover, the predictive force of lockdown obtained for each measure of centrality increases with larger values of *λ*.

#### Robustness

In Table G2 in Online Appendix G, we provide robustness checks for other correlations between the four network metrics and average optimal lockdown probabilities in the lattice, random, and scale-free networks. We observe that all other centrality measures are positively correlated with the average optimal lockdown probabilities, apart from the lattice network. Also, in line with the small-world network, the degree centrality appears to have a stronger correlation with the lockdown in the random and scale-free networks. Though the correlation between the network metrics and optimal lockdown probabilities becomes stronger as the tolerable infection incidence increases in small-world and scale-free networks, the direction of the changes is non-monotonic in lattice and random networks. The latter simulation results suggest that we should be cautious about making any conclusions about the sign and direction of the relationship between the tolerable infection incidence, *λ*, the network centrality measures, and the optimal lockdown probabilities. Nevertheless, the simulation results in Table 1 and in Online Appendix G (Tables G2 and G3) imply that in a society organized as either a small-world network or a scale-free network, with a higher level of tolerance for the virus, more central individuals will suffer fewer deaths as a result of being more severely isolated. In Section 5, we use data from the network of US nursing and long-term care homes (Chen et al. 2021) to test some of these simulation results.

#### Remark

Intuitively, though a full lockdown may be a solution in a pure epidemiological model, it cannot be optimal in our N-SIRD model because the goal is to disconnect the contact network while maintaining economic activities. It follows that under our optimal lockdown policy, not all agents would be in the lockdown. This analysis highlights the limitations of quasi-universal lockdown policies such as those implemented in several countries worldwide in the early period of COVID-19. Our policy recommendations are consistent with studies and reports suggesting shutting down only particular sectors of society during a pandemic like COVID-19 (see, e.g., Acemoglu et al. 2021, Bosi et al. 2021, Chang et al. 2021, and ; Pestieau and Ponthière 2022). Specifically, lockdowns should target sectors that serve as social and economic hubs and attract large numbers of individuals, such as large shopping centers, airports and other public transportation infrastructures, schools, certain government buildings, entertainment fields, parks, and beaches.

## Empirical application

In this section, we calibrate our N-SIRD model, estimate the tolerable COVID-19 infection incidence level for 26 US states, and test some of our model’s predictions using unique data on networks of the US nursing homes and long-term care facilities.

### Relevance

The example of US nursing home networks is a relevant test of our theoretical model for two main reasons. First, the senior population (adults 65 and older) accounts for a significant share of COVID-19 deaths in the USA. As of September 24, 2021, seniors account for 16% of the US population but 77.9% of US COVID-19 deaths (Yang 2021). Nursing and long-term care facilities have been at the center of many COVID-19 outbreaks, and this situation led the US federal government to ban nursing home visits on March 13, 2020. This restriction has enabled researchers from the “Protect Nursing Homes” project to construct a network of physical contacts in US nursing homes, using geolocation data for 50 million smartphones. They observed that 5.1% of smartphone users (approximately 501,503 staff and contractors) who visited a nursing home for at least 1 h also visited another facility during the 11-week study period, even after visitor restrictions were imposed. The ban on nursing home visits—an example of a lockdown policy to reduce contagion in nursing homes—created an environment where the network of contacts was the primary source of virus spread. Second, as we explain in Section 4, the calibration of production functions for senior care services in each US state can be viewed as a representation of a simple production environment in the optimal control problem of our theoretical model.

### Capturing the tradeoff between saving lives and economic prosperity

The main exogenous constraint introduced in the theoretical model, the tolerable infection incidence level *λ*, reveals the extent to which governors in different US states are willing to curb the spread of SARSCOV-2, the virus that causes COVID-19. In order words, *λ* captures the governor’s tradeoff between health and wealth. A high value for *λ* is equivalent to a “laissez-faire” policy, indicating a planner’s inclination to maximize economic gains even if this theoretically results in more infections and deaths. Section 5.1 estimates the values of *λ* for 26 US states. Furthermore, since COVID-19 responses have been highly politicized in the USA and given the large heterogeneity in the values of *λ*, we investigate how political ideology (measured by the party of the governor) and other state-specific factors determine *λ*.^15^ Section 5.2 uses the estimated values of *λ* to test some theoretical predictions of our N-SIRD model with lockdown. Precisely, we explore whether the simulation results are consistent with reality. For instance, we examine whether “laissez-faire” policies lead to more deaths. We also investigate the effect of network centrality and the tolerable infection incidence on COVID-19 death in nursing facilities.

### Estimation of COVID-19 tolerable infection incidence

#### Data, calibration, and estimation

To calibrate our parameter of interest, we use data from several sources. Data on the economic variables come from the Bureau of Labor Statistics and the Senior Living. project.^16^ Data on the US nursing home networks were obtained from Chen et al. (2021). We obtain the calibration of the epidemiological parameters from Statista.^17^ Using the data on US nursing home networks, we calibrate a nursing home’s production function; for more details on this calibration, we refer to Online Appendix F.1. We describe in Table 2, all sources of calibrated and estimated parameters for each US state, which we use in our empirical application.

##### US nursing home networks

**Fig. 5:**
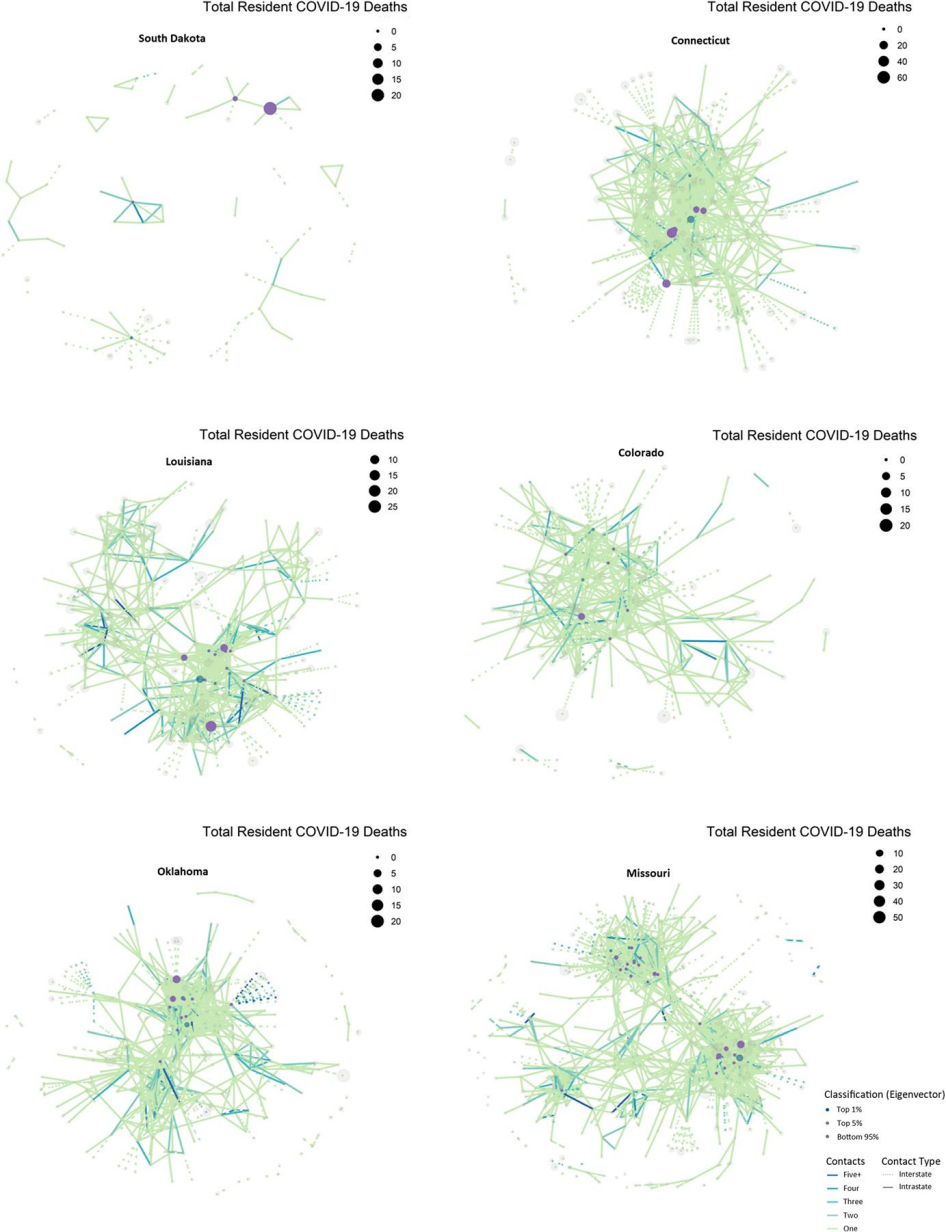
Nursing home network structures in South Dakota, Connecticut, Louisiana, Colorado, Oklahoma, and Missouri. Notes: Details for each network configuration are provided in Table 3. In the network, node size varies with the number of COVID-19 deaths among residents reported to the US Centers for Medicare & Medicaid Services as of May 31, 2020; edge color differs with the number of contacts between nursing homes; a solid (resp. dotted) edge line corresponds to a connection between two nursing homes within the same US state (resp. in two different states); and node color differences are based on eigenvector ranking, with the dark red color, for example, highlighting the top 1% of facilities with high eigenvector centrality in the network

We consider each nursing home as a node in the transmission network. Two nursing homes are connected if the same smartphone signal is recorded in both homes’ locations. The number of distinct signals recorded gives a weight to the connection or link between two nursing homes. Nursing and long-term care facilities display a wide range of connectedness with other facilities. Chen et al. (2021) use different network metrics to predict COVID-19 in nursing homes. In this empirical application of our N-SIRD model, we focus on the eigenvector centrality, which measures the extent to which a nursing home in a US state is connected to other highly connected nursing homes in the state.^18^ To illustrate how the eigenvector centrality measure differs across nursing homes, we present network graphs for a subset of homes in six states as depicted in Fig. 5 and summarized in Table 3. More-connected nursing facilities are generally toward the center of each graph, and facilities with fewer contacts are on the periphery. Table 4 summarizes the descriptive statistics of US nursing homes. We refer to Chen et al. (2021) for additional details on nursing homes characteristics and network centrality measures in these care facilities.

##### Estimation of *λ*

**Fig. 6:**
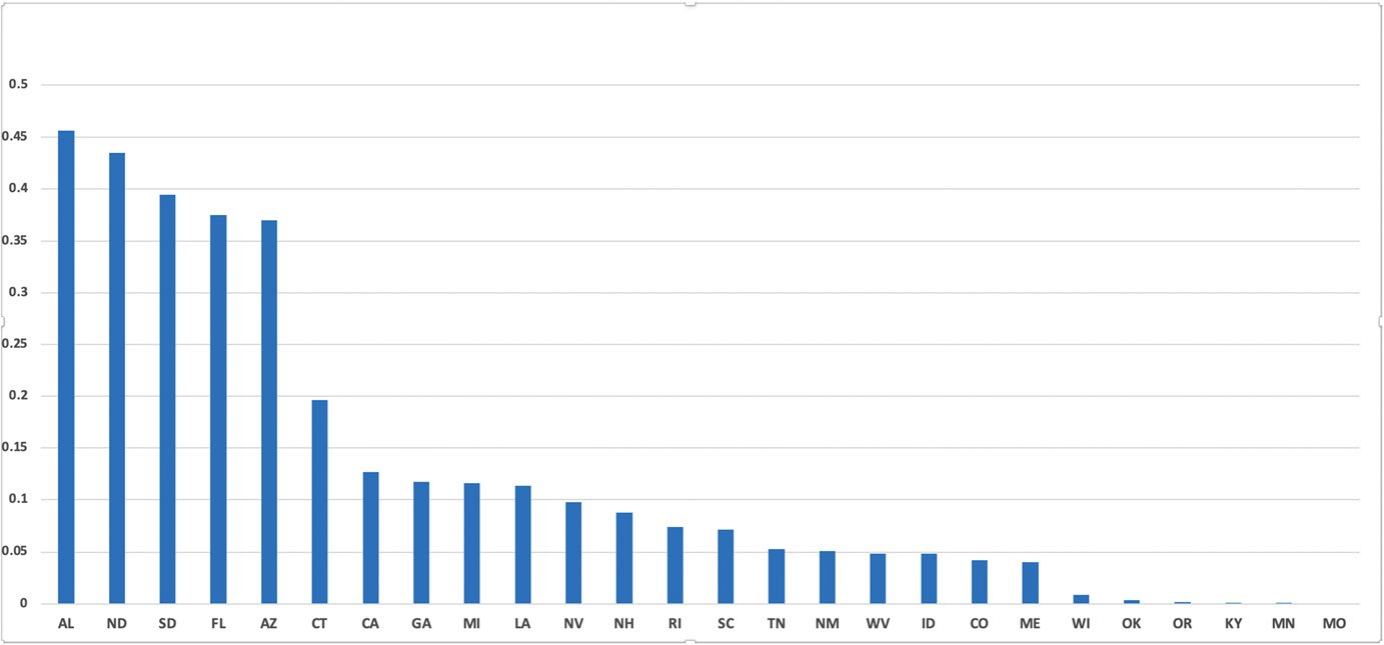
US states’ tolerable COVID-19 infection incidence levels *λ*. Notes: The parameter *λ* estimates the tolerable COVID-19 infection incidence of the US state governor from May 31 to August 16, 2020. Using the data and the N-SIRD model with lockdown, we estimate *λ* for 26 US states. The average value of *λ* is 0.12 and its standard deviation is 0.13

 We estimate the parameter measuring the governor’s tolerable infection incidence using a simulated minimum distance estimator (Gertler and Waldman 1992; Forneron and Ng 2018). Indeed, for each potential value of *λ*, the planner’s problem is solved and the dynamics of death in the model over 77 days is compared to raw data on elderly death dynamics provided by the New York Times death count from May 31 to August 16, 2020. The value of *λ* that will minimize the distance between the two dynamics will be the estimate of the tolerable infection incidence level for that US state’s governor. In Online Appendix F.2, we provide additional explanation on estimating *λ*. The procedure is carried out for 26 US states and the estimate values of *λ* are displayed in Fig. 6. For each of the 26 US states, the tolerable infection incidence level *λ* is significantly different from zero. The estimated tolerable infection incidence levels range from 0.0006 for the state of Missouri (MO) to 0.45 for Alabama (AL). The average value of *λ* is 0.12 and the standard deviation is 0.13 indicating a substantial level of dispersion. We investigate in Section 5.1.2, the possible sources of such heterogeneity.

#### Origins of the tolerable infection incidence heterogeneity

Whether it is about economic lockdowns, mask mandates, or COVID-19 vaccines, the public debates in the USA have been divided along political lines (Adolph et al. 2021; Neelon et al. 2021). The extent to which this division has affected the COVID-19 pandemic is at the heart of a new and growing literature. We contribute to this literature by examining whether the party affiliation of a US state’s governor predicts the tolerable COVID-19 infection incidence. We regress the tolerable infection incidence level on the party affiliation of the state governor in the period covered by the sample (May 31 to August 16, 2020) and other controls. This regression sheds light on the most critical determinants of the US state’s choice of the tolerable COVID-19 infection incidence level. The analysis also represents an attempt to validate our estimation of the parameter *λ* using information from external sources.

##### Regression results

The estimation results shown in Table 5 indicate that Democratic governors have a tolerable infection incidence that is 8% lower than that of their Republican counterparts. Thus, Republican governors are more inclined to implement “laissez-faire” policies, which mirrors the traditional pro-market position of the party. This statement is in line with Baccini and Brodeur (2021), who find consistent results on the role of political ideology in the response of US states to the COVID-19 pandemic. For instance, their results suggest that during the early COVID-19 epidemic, Democratic governors emphasized health and safety and were significantly more likely to implement a statewide stay-at-home order. By contrast, Republican governors were particularly concerned about the economic costs of stay-at-home measures and were less likely to implement those policies. Unsurprisingly, we find there is a positive association between the number of deaths in a US state’s nursing homes and tolerable COVID-19 infection incidence level in that state. However, having a higher number of deaths in nursing homes seems to reduce the gap in the tolerable infection incidence between Republican and Democratic governors, as illustrated by estimates in columns (2) to (4) of Table 5. Governors from different parties therefore tend to converge in their policies when faced with a high death count. The estimation results also suggest that the gender of the governor has an effect on the tolerable infection incidence level, with this level being higher in female governors by about 7%. Moreover, being located in the South increases the tolerable infection incidence level by 5%.

In summary, our analysis suggests that both the ideological orientation of a state’s governor and the statewide severity of the pandemic impact the choice of the tolerable COVID-19 infection incidence across the 26 US states sampled. Additionally, we find the gender of a US state’s governor, as well as a state’s geographic location as essential determinants of tolerable COVID-19 infection incidence level.

### Testing some N-SIRD model’s predictions

In our empirical analysis, we estimate the following linear model:

5 $$\begin{array}{@{}rcl@{}} covid\_death_{ijs} &=& a_{0} \lambda_{s} + a_{1} Eig\_Cent_{ijs}+ a_{2} County\_ssa_{js} +a_{3} D\_Profit_{ijs} \\ &+& b_{1} \lambda_{s}\times Eig\_Cent_{ijs} + b_{2}\lambda_{s}\times County\_ssa_{js}+b_{3} \lambda_{s}\times D\_Profit_{ijs} \\ &+& c^{\prime}X_{ijs}+ \theta_{j} + \varepsilon_{ijs}, \end{array}$$

where *covid*_*death*_*ijs*_ is a variable counting the total number of COVID-19 deaths in nursing home *i*, in county *j* and US state *s*; *λ*_*s*_ is the tolerable infection incidence in US state *s*; *Eig*_*Cent*_*ijs*_ is the eigenvector centrality index for the nursing home; *County*_*ssa*_*js*_ is the county *j*’s average socio-economic status; *D*_*Profit*_*ijs*_ is an indicator for whether nursing home *i* is for profit (1 if for-profit, and 0 otherwise); *X*_*ijs*_ represents other exogenous characteristics of the nursing home including the constant; and *𝜃*_*j*_ is the county fixed effect.^19^ The parameters of interest are *a*_0_, *a*_1_, *a*_2_, *a*_3_, *b*_1_, *b*_2_, and *b*_3_. The estimated values of these parameters can be found in Table 6. Estimating the tolerable infection incidence level in each US state allows us to verify some of the model’s predictions.

#### Tolerable infection incidence and COVID-19 death

Figure 1d in Section 4.1 illustrates the relationship between the tolerable infection incidence level and the death dynamics. This statics comparative analysis implies that a more higher value for *λ* is associated with more COVID-19 deaths. The OLS estimation results in Table 6 suggest that there is a positive association between the tolerable infection incidence level and the total number of COVID-19 deaths in a nursing home. A five standard-deviation increase in the tolerable infection incidence is expected to lead approximately to one additional death in a nursing home everything else being equal. This means that laissez-faire policies will result in more COVID-19 deaths. This result remains robust after controlling for county fixed-effects, the level of income of a nursing home’s residents (as proxied by the average socio-economic status in the county), the quality of care provided, and whether the nursing home operates on a for-profit basis.

#### Tolerable infection incidence, network centrality, and COVID-19 death

The simulation results uncovered in Section 4 suggest that the level of network centrality plays a pivotal role in the choice of optimal lockdown and the diffusion of an epidemic that spread through networks. The optimal lockdown policy targets more central individuals with a higher probability. Table 1 in Section 4.3 suggests that higher values of tolerable infection incidence levels are associated with a higher likelihood of lockdown for central agents in a network. Therefore, our simulation would predict that adopting a laissez-faire approach (i.e., increase in *λ*) will reduce the impact of network centrality on the number of COVID-19 deaths because more central individuals are likely to be sent into lockdown. In other words, under a laissez-faire policy, the difference in the number of deaths between central and peripheral nursing homes is reduced. The regression results in Table 6 validate this prediction. Column (1) shows that being more central is associated with more COVID-19 deaths in the nursing homes. Column (2) shows the interaction between eigenvector centrality and the tolerable infection incidence. The interaction term has a negative and statistically significant effect on total COVID-19 deaths. An increase in the level of the tolerable infection incidence therefore reduces the relative death toll of more central nursing homes. Columns (2) and (5) of Table 6 show the robustness of this result to the introduction of several controls. We also verify another prediction of our model’s simulation in the sample under investigation. Our results complement Chen et al. (2021) by showing that, while the level of eigenvector centrality matters in the propagation of the epidemic and death count, there exists heterogeneity in the extent of its relevance. More precisely, we show that the social planner’s tolerable infection incidence affects the relationship between the level of centrality and the number of COVID-19 deaths. This relationship is less pronounced under a laissez-faire regime.

#### Tolerable infection incidence and wealth accumulation

The simulations in Section 4 also show the relationship between the tolerable incidence and economic performance. Figure 1c suggests that more laissez-faire policies are associated with a lower total economic cost. The estimation results in Table 8 put this prediction to a test by estimating the effect of US states’ tolerable COVID-19 infection incidence on their level of GDP growth in 2020. We present in Table 7, the descriptive statistics of GDP and US states’ governorship political affiliation and gender in 2020. In accordance with the theoretical simulations, our estimation results suggest a positive relationship between *λ* and the GDP growth. The effect of the tolerable infection incidence on economic growth is larger for Republican governors. These results are robust to the inclusion of controls for regional differences and the gender of the governor.

#### Additional regression results

We also assess how laissez-faire policies affect the relationship between the COVID-19 death toll, economic conditions, and type of nursing home (for-profit or not). Column (5) in Table 6 shows that laissez-faire policies more negatively affect nursing homes in economically deprived counties. Our analysis also shows that for-profit nursing homes have 27% more deaths compared to not-for-profit nursing homes (see columns (1) to (3) in Table 6). Moreover, the type of the nursing home and the tolerable infection incidence are the main drivers of the difference in COVID-19 deaths in nursing homes. Indeed, when we introduce the interaction term between *λ* and for-profit (D_Profit) in column (4), both the effects of *λ* and the for-profit indicator (D_Profit) become smaller in absolute value and statistically insignificant; only the interaction term has a positive and statistically significant coefficient, meaning that the detrimental effects of laissez-faire policies are primarily present in nursing homes that operate on a for-profit basis. We also note that better rated nursing homes have significantly less deaths.

#### Summary

The findings of Table 8 validate some essential predictions of the N-SIRD model using data from nursing home networks in 26 US states. Indeed, we provide evidence suggesting that a higher tolerable infection incidence is associated with more COVID-19 deaths. Moreover, centrality plays an essential role in optimal lockdown, and laissez-faire policies significantly interact with network centrality. We also show that the tolerable infection incidence seems to mediate the impact of economic variables on the human cost of the pandemic. The existence of a positive correlation between the tolerable infection incidence and economic performance is tested and validated in our sample.

## Concluding remarks

This study addresses the problem of finding an optimal lockdown policy during a pandemic for a social planner who *prioritizes* health over short-term economic gains. Agents are connected through a weighted network of contacts, and the planner’s objective is to determine the policy that contains the spread of infection below a *tolerable* incidence level and maximizes the present discounted value of real income, in that order of priority. We formalize this tradeoff by using lockdown as a policy instrument in an optimal control problem that mixes an individual mean-field epidemiological model and a simple production environment.

Our analysis reveals that the planner’s optimal lockdown policy depends on tolerable infection incidence level and social network structure. Using simulation-based comparative statics analyses in combination with early COVID-19 data, the paper highlights the crucial role of network structure in infection spread. Mainly, it quantifies the tradeoff between the tolerable infection incidence and human losses on the one hand and the economic losses due to the pandemic on the other hand. The simulation exercises also show how different network centrality measures correlate with individual lockdown probabilities and how this correlation varies with the tolerable infection incidence level.

We use unique data on US nursing home networks, as well as other data sources, to calibrate our model and estimate the tolerable COVID-19 infection incidence level (*λ*) for 26 US states. Our estimates show significant variation in *λ* across US states. This variation is partly explained by COVID-19 fatalities, the gender of a state’s governor, the party affiliation of a state’s governor, and states’ geographic location. Using these estimated values of *λ*, we find that policies that tolerate more virus spread (laissez-faire) are associated with an increased number of deaths in nursing homes and an increase in a state’s GDP growth. We also find significant interactions between *λ* and other essential variables. In particular, we find that laissez-faire policies are more harmful to nursing homes that are more peripheral in networks. Additionally, laissez-faire policies are also more detrimental to nursing homes in deprived counties and those operating on a for-profit basis. These latter findings are relevant and valid for organizations that seek to maximize economic gains.

**Table 1:** Network centrality and lockdown probability in a small-world network

*λ*	Degree		Closeness		Betweenness		Eigenvector	
	Corr	*p*-value	Corr	*p*-value	Corr	*p*-value	Corr	*p*-value
0.1	0.36	8e-33	0.34	9e-29	0.33	3e-27	0.29	1e-20
0.05	0.25	5e-16	0.21	1e-11	0.21	6e-12	0.17	1e-07
0.01	0.26	1e-16	0.18	4e-09	0.18	3e-09	0.13	4e-05

Table 1 illustrates the correlation (corr) between measures of centrality and average optimal lockdown probability in a small-world network for three values of *λ*. The *p*-value for each centrality measure is for the test of the hypothesis *H*_0_ *ρ* = 0 vs *H*_1_ *ρ*≠ 0. In Table G2 in Online Appendix G, we replicate Table 1 for scale-free, random, and lattice networks. We also replicate Table 1 using recent epidemiological data of the COVID-19 Delta variant (see Table G3 in Online Appendix G). We find that the simulation results in Table G2 are qualitatively consistent with the findings in Table 1

**Table 2:** Description and sources of calibrated and estimated parameters for each US state

Parameters or variables	Values	Definitions and sources	Utilization
Epidemiological			
β	*R*_0_/18	The COVID-19 reproduction numbers *R*_0_ estimated during April to July 2020, from Statista	Calibration
*γ*	(1-death/case)/18	Case and death per 1000 in nursing homes in each US state as of Sep. 2020 from Statista	Calibration
*κ*	(Death/case)/18	Case and death per 1000 in nursing homes in each US state as of Sep. 2020 Statista	Calibration
Death count	80% of COVID-19 death	New York Times in each US state from May 31 to August 16, 2020	Calibration
*A*	Network of nursing homes	Protect Nursing Home project	Calibration
Economic			
Price	Average hourly cost of a private room	Senior Living project	Calibration
Wage	Average hourly wage by state	BLS calibration	
*α*	Cobb-Douglass production function	Replication data from Chen et al. (2021) estimation for each state	Calibration
Regressions tables	variables	Replication data from Chen et al. (2021) and authors’ calculation	Estimations
Capital	Number of beds in the nursing home	Replication data from Chen et al. (2021)	Calibration

**Table 3:** Network characteristics for six selected US nursing home networks

States	Number of	COVID-19 deaths			Eigenvector centrality	
	nursing homes	Max	Mean	Sd	Mean	Sd
South Dakota	103	22	0.22	2.17	0.043	0.17
Connecticut	196	67	7.31	10.46	0.13	0.21
Louisiana	259	26	2.68	5.06	0.09	0.22
Colorado	214	22	1.49	3.73	0.11	0.18
Oklahoma	257	17	0.3	1.59	0.08	0.18
Missouri	483	21	0.56	2.52	0.07	0.15

Data comes from Chen et al. (2021). COVID-19 deaths are confirmed among residents reported to the US Centers for Medicare and Medicaid Services (CMS) as of May 31, 2020. “Sd” means standard deviation

**Table 4:** Descriptive statistics of US nursing homes

Variable	Mean (standard deviation)
COVID-19 information	
Cases	84.47 (237)
Death	1.84 (5.94)
Network metrics	
Home degree centrality	6.21 (7.83)
Home eigenvector centrality	0.08 (0.18)
Regulatory measures	
For profit	0.703
Number of beds	105.61 (59.04)
Number of beds occupied	76.97 (48.01)
CMS quality rating (1–5)	3.69 (1.24)
County SSA	391.39 (273.53)
Number of nursing homes	15277

Data are from Chen et al. (2021). Binary variables are percent of nursing homes; continuous variables are mean values, with standard deviations in parentheses

**Table 5:** Origins of the tolerable COVID-19 infection incidence heterogeneity

	(1)	(2)	(3)	(4)
Republican governor	0.0973^∗∗∗^	0.104^∗∗∗^	0.0999^∗∗∗^	0.0756^∗∗∗^
	(33.06)	(33.28)	(31.93)	(20.59)
Republican × Covid_Death		–0.00293^∗∗∗^	–0.00376^∗∗∗^	–0.00351^∗∗∗^
		(–5.72)	(–7.12)	(–6.84)
Covid_Death		0.00280^∗∗∗^	0.00291^∗∗∗^	0.00289^∗∗∗^
		(11.00)	(10.48)	(9.61)
Female governor			0.0536^∗∗∗^	0.0693^∗∗∗^
			(11.15)	(14.17)
South				0.0514^∗∗∗^
				(12.61)
Constant	0.174^∗∗∗^	0.0718^∗∗∗^	0.0656^∗∗∗^	0.0553^∗∗∗^
	(63.55)	(63.14)	(49.25)	(33.09)
Observations	6985	6564	6564	6564
*R* ^2^	0.128	0.138	0.158	0.183

The dependent variable is the US state’s tolerable COVID-19 infection incidence (*λ*). Standard errors are robust to heteroscedasticity of unknown form. ^∗^ *p* < 0.1; ^∗∗^ *p* < 0.05; ^∗∗∗^ *p* < 0.01; *t* statistics are in parentheses

**Table 6:** Estimation of the effects of laissez-faire policies (*λ*) on number of deaths in US nursing homes

	(1)	(2)	(3)	(4)	(5)
*λ*	0.713^∗∗^	1.063^∗∗∗^	2.127^∗∗∗^	–0.105	1.573^∗∗^
	(2.04)	(3.19)	(3.25)	(–0.21)	(2.12)
Eig_Cent	1.006^∗∗∗^	1.482^∗∗∗^	1.026^∗∗∗^	1.011^∗∗∗^	1.533^∗∗∗^
	(3.27)	(3.98)	(3.35)	(3.29)	(4.11)
County_ssa	–0.000780	–0.000824	–0.000521	–0.000793	–0.000584
	(-1.09)	(–1.15)	(–0.72)	(–1.11)	(–0.81)
D_Profit	0.266^∗∗^	0.268^∗∗^	0.269^∗∗^	0.101	0.0836
	(2.28)	(2.29)	(2.30)	(0.73)	(0.60)
*λ* × Eig_Cent		–3.944^∗∗^			–4.157^∗∗^
		(–1.97)			(–2.08)
*λ* × County_ssa			–0.00446^∗∗^		–0.00445^∗∗^
			(–2.47)		(–2.48)
*λ* × D_Profit				1.231^∗∗^	1.387^∗∗^
				(1.97)	(2.20)
Overall_Rating	–0.207^∗∗∗^	–0.207^∗∗∗^	–0.207^∗∗∗^	–0.210^∗∗∗^	–0.210^∗∗∗^
	(–5.07)	(–5.07)	(–5.06)	(–5.13)	(–5.12)
County FE	Yes	Yes	Yes	Yes	Yes
Observations	6478	6478	6478	6478	6478
*R* ^2^	0.072	0.073	0.073	0.073	0.074

Standard errors are robust to heteroscedasticity of unknown form; *t* statistics in parentheses. ^∗^ *p* < 0.1; ^∗∗^ *p* < 0.05; ^∗∗∗^ *p* < 0.01. In Table G4 in Online Appendix G, we show that our main empirical results in Table 6 are robust when replacing eigenvector centrality by the degree centrality

**Table 7:** Descriptive statistics of GDP and US state governorship political affiliation and gender in 2020

	Mean	Standard deviation	Minimum	Maximum
GDP growth, %	–3.46	1.47	–7.00	–0.10
Democrat governor	0.47	0.50	0.00	1.00
Female governor	0.18	0.39	0.00	1.00

**Table 8:** Estimation of the effects of laissez-faire policies on US state’s GDP growth in 2020

	(1)	(2)	(3)	(4)	(5)
*λ*	3.812^∗∗^	4.290^∗∗^	5.401^∗∗^		
	(2.57)	(2.15)	(2.35)		
Democrat governor		0.0554	0.845		–2.040^∗∗^
		(0.08)	(1.02)		(–2.22)
Female governor		–0.513	–0.765		–0.593
		(–0.91)	(–1.31)		(–0.96)
South		–1.083^∗^	–1.124^∗^		–1.261^∗^
		(–1.78)	(–1.92)		(–2.06)
Democrat × *λ*			–10.15^∗^		
			(–1.95)		
*log*(*λ*)				0.169	0.420^∗∗^
				(1.47)	(2.25)
Democrat × *log*(*λ*)					–0.540^∗∗^
					(–2.38)
Constant	–4.100^∗∗∗^	–3.674^∗∗∗^	–3.804^∗∗∗^	–3.088^∗∗∗^	–1.700^∗^
	(–11.87)	(–5.16)	(–5.32)	(–6.57)	(–2.05)
Observations	26	26	26	26	26
*R* ^2^	0.165	0.307	0.382	0.057	0.320

Standard errors are robust to heteroscedasticity of unknown form. ^∗^ *p* < 0.1; ^∗∗^ *p* < 0.05; ^∗∗∗^ *p* < 0.01; *t* statistics are in parentheses

## Supplementary Information

Below is the link to the electronic supplementary material.

Supplementary file1 (PDF 1.19 MB)

## Footnotes

1 Our assumption that health is prioritized over short-term economic prosperity is consistent with recent observations, e.g., Heap et al. (2020) and Stiglitz (2020). For instance, Stiglitz wrote that: “There can be no economic recovery until the virus is contained, so addressing the health emergency is the top priority for policymakers.” (n.d ; Stiglitz 2020).

2 We refer to Pastor-Satorras et al. (2015) for an excellent survey on these models.

3 See, e.g., Pindyck (2020) and Bosi et al. (2021) for the expression of a similar concern. In the same perspective, Pestieau and Ponthière (2022) examine how to design optimal lockdown strategies under different social welfare criteria.

4 *R*_0_ represents the expected number of secondary infectious produced by a primary case introduced in a fully susceptible population (Anderson and May 1992).

5 Our series of robustness checks conjecture that the simulation results with lattice, random, and scale-free networks are qualitatively consistent with those obtained with the small-world network.

6 We refer the reader to Section 5.1.1 for other data sources.

7 Recent previous approaches include, among others, Karaivanov (2020), who examines the transmission of COVID-19 through a dynamic social network model embedding the SIR model onto a graph of network contacts; Bethune and Korinek (2020), who study the externalities associated with public health interventions in susceptible-infected-susceptible (SIS) and SIR models of infectious disease; Acemoglu et al. (2021), who propose a multi-risk SIR model; Berger et al. (2022), Federico and Ferrari (2020), Gollier (2020b), Prem et al. (2020), Alvarez et al. (2021), Bandyopadhyay et al. (2021), Bisin and Moro (2021), Eichenbaum et al. (2021), and Ma et al. (2021), who analyze optimal non-pharmaceutical controls in SIR models; Chang et al. (2021), who use Google mobility data to construct mobility network and metapopulation susceptible–exposed–infectious–removed (SEIR) models to explain differences in COVID-19 fatalities and to inform reopening decisions in ten large US metropolitan areas; and Harris (2020) and Kuchler et al. (2021), who document the importance of social media networks (e.g., Facebook) in the selection of targeted lockdown policies.

8 We thank an anonymous referee for bringing this issue to our attention.

9 Some studies exploring virus spread in networks consider that nodes with the same number of connections are equivalent. Then, instead of working with quantities specifying the state of each node *i* ∈ *N* (as we do throughout), the relevant variables specify the state of all nodes with the same number of connections. For more details, we refer to a review of degree mean-field models by Pastor-Satorras et al. (2015). Other recent studies consider partitioning individuals into different risk groups following age or stage structured compartmental epidemiological models, e.g., Acemoglu et al. (2021) and Pestieau and Ponthière (2022).

10 Our assumption of constant transmission rates β, *γ*, and *κ* follows the canonical SIR and SIRD epidemiological models. Recent studies apply these models to analyze the possible COVID-19 outcomes, e.g., Anastassopoulou et al. (2020), Acemoglu et al. (2021), and Alvarez et al. (2021). We note that, unlike our framework which considers a fixed contact rate β, other studies tend to use time-varying contact rates when examining optimal public health interventions, such as lockdown and social distancing policies, e.g., Gollier (2020b).

11 Our assumption of full effectiveness is contrary to Alvarez et al. (2021) who consider the case where full lockdown is only partially effective in eliminating the transmission of the virus. Alvarez et al. justify this limitation by the fact that people can still interact in full lockdown. We assume that being in full lockdown severs the agent’s contacts with all neighbors in the prevailing network.

12 Assuming an irreversible lockdown under a tractable epidemiological model enables the researcher to derive a closed-form solution while establishing the convexity of the problem with second-order conditions (Seierstad and Sydsaeter 1986; Bosi et al. 2021). As in Alvarez et al. (2021), the interactions between dynamic lockdown policies and our N-SIRD epidemiological model may make the planner’s problem non-convex. Therefore, we cannot use second-order conditions to verify whether a given candidate policy is an optimal solution. In other words, it would not be possible to prove that our optimal lockdown policy is indeed minimizing the economic costs of lockdown. Though we do not address the convexity issue, we follow Alvarez et al. (2021) and Acemoglu et al. (2021) and use simulations to conduct comparative statics analyses within our framework. In addition, we provide an empirical application of the simulation results.

13 In our empirical analysis, discussed in Section 5, the network of nursing homes in each US state is not necessarily binary. Instead, *A*_*i**j*_ ranges from 1 to 832 contacts, where *i* and *j* represent two distinct nursing homes. Since our empirical findings appear to be consistent with the simulation results, we believe that the binary nature of the network structure does not affect the qualitative dimension of our findings. In the planning problem, we choose values for the parameters of the production function that do not match those obtained from our data, for the purpose of illustration. Thus, one should interpret the quantitative outcomes of our model with caution. Nevertheless, in our empirical application, discussed in Section 5, we choose these parameters to match our US state-level data as closely as possible.

14 Based on the simulation results (Fig. G4 in Online Appendix G) that we obtain by replicating Fig. 1 with the COVID-19 Delta variant information, we conjecture that a similar exercise with the lattice, random, and scale-free network structures would yield consistent results.

15 Neelon et al. (2021) suggest that there is an association between a governor’s party affiliation and COVID-19 infections and deaths (also, see, e.g., Baccini et al. 2021 and Chen and Karim 2021 for additional evidence linking political party of leaders and COVID-19 fatalities). We complement these findings by investigating the association between the COVID-19 estimated tolerable infection incidence and the governor’s party affiliation. More importantly, we view our analysis as an “external” validation of our estimation of *λ*; indeed, it follows from the aforementioned studies that *λ* should be higher for states governed by Republicans than for states governed by Democrats.

16 We gathered information from the Senior Living project on 9/9/2021 at https://www.seniorliving.org/nursing-homes/costs/.

17 This data is available at https://www.statista.com. Statista provides information on the reproduction number for COVID-19, and the COVID-19 infection and death rates among nursing home residents in each US state as of September 2020.

18 Table G4 in Online Appendix G shows that our main empirical results are robust when replacing eigenvector centrality by the degree centrality.

19 The choice of the total number of COVID-19 deaths rather than cases, as the outcome variable, is motivated by two reasons. First, the number of COVID-19 cases contains both asymptomatic patients and those who will later recover, so it cannot be an appropriate measure of the human cost of the pandemic. Second, as represented in Fig. 1b, depending on the point in time during the pandemic, there may be no difference in the number of infected individuals as a function of the tolerable infection incidence. On the contrary, the total number of deaths displays unambiguous dynamics which makes the theoretical predictions of our N-SIRD model easier to test. In addition, the total number of deaths is unambiguously a proxy for the human cost of the pandemic.
