# Supplementary material for: Optimal interventions in networks during a pandemic
Source: J Popul Econ. 2022 Aug 13;36(2):847–83. doi: 10.1007/s00148-022-00916-y (PMC9375093; doi:10.1007/s00148-022-00916-y)
Supplement: Supplementary file 1 — Supplementary file1 (PDF 1.19 MB) [file 148_2022_916_MOESM1_ESM.pdf]

Online Appendix to "Optimal Interventions in Networks during a Pandemic"

Roland Pongou[1], Guy Tchuente[2*†] and Jean-Baptiste Tondji[3†]

[1]Department of Economics, University of Ottawa, 120 University Private, Social Sciences Building, Ottawa, K1N 6N5, Ontario, Canada.
[2]School of Economics, University of Kent, Kennedy Building, Park Wood Road, Canterbury, CT2 7FS, Kent, United Kingdom.
[3*]Department of Economics, The University of Texas Rio Grande Valley, 1201 West University Drive, Edinburg, 78539, Texas, United States.

*Corresponding author(s). E-mail(s): g.tchuente@kent.ac.uk;
Contributing authors: rpongou@uottawa.ca;
jeanbaptiste.tondji@utrgv.edu;
†These authors contributed equally to this work.

Appendix A The final size of the pandemic

To reflect the impact of the epidemic in a totally susceptible population, we set $s_i(0) \approx 1$ and assume following Andreasen (2011) that $x_i(0)$ is positive with $x_i(0) \approx 0$ for all $i \in N$. We also follow Brauer (2008) by stating that $x_i(t) \to 0$ when $t \to \infty$, while there exists some real number $s_i(\infty)$ such that $s_i(t) \to s_i(\infty)$ when $t \to \infty$. The first two equations in the system (ODE) can be rewritten as:

$$\begin{cases} \dot{x_i} = \beta s_i \sum_{j \in N} [A_{ij}(1-l_i)(1-l_j)x_j] - (\gamma + \kappa)x_i \\ \dot{s_i} = -\beta s_i \sum_{j \in N} [A_{ij}(1-l_i)(1-l_j)x_j]. \end{cases}$$

We determine the value of $s_i(\infty)$ by integration of these equations over the entire epidemic period, which entails

$$\log\, s_i(\infty) - \log\, s_i(0) = -\beta \sum_{j\in N} [A_{ij}(1-l_i)(1-l_j) \int_0^\infty x_j d_t], \tag{A1}$$

$$s_i(\infty) - s_i(0) = \int_0^\infty (\dot{x_i} + \dot{s_i})dt = -(\gamma+\kappa) \int_0^\infty x_i d_t. \tag{A2}$$

We derive the outcome of the epidemic in terms of the ratio $\sigma_i = \frac{s_i(\infty)}{s_i(0)}$, which is approximately the probability of being susceptible and remaining uninfected at the end of the epidemic, $s_i(\infty)$, given that $s_i(0) \approx 1$. We use the column vector $\sigma = (\sigma_1, ..., \sigma_n)^T$ to express the size of the epidemic since the infected rate for agent i is $z_i = 1 - \sigma_i$, and the final size of the epidemic in the whole population is $\sum_{j\in N} z_j N$. For each agent i, the attack rate z_i also equals $r_i(\infty) + d_i(\infty)$, since $x_i(t) \to 0$ when $t \to \infty$. Noting that $(1-\sigma_i)s_i(0) = s_i(0) - s_i(\infty)$, and substituting Eq.(A2) into Eq.(A1) yields the size of epidemic σ as a solution of the coupled implicit equations:

$$0 = \log\, \sigma_i + \sum_{j\in N} [\frac{\beta}{\gamma+\kappa} A_{ij}(1-l_i)(1-l_j)s_j(0)](1-\sigma_j) = \mathcal{H}_i(\sigma),\ i = 1, 2..., n. \tag{A3}$$

Recall that $\mathcal{M}_{ij}$, which is the (i,j)-entry in the next-generation matrix is $\frac{\beta}{\gamma+\kappa} A_{ij}(1-l_i)(1-l_j)$. With $s_i(0) \approx 1$ for all i, the final size equation (A3) can be written in matrix notation with the next-generation matrix $\mathcal{M}$, the coordinate-wise log-function, and the column null vector $\overline{0} = (0, ..., 0)^T$ as:

$$\overline{0} = \log\, \sigma + \mathcal{M}(\overline{1} - \sigma) = \mathcal{H}(\sigma), \tag{A4}$$

where $\overline{1} = (1, ..., 1)^T$. Now, taking the coordinate-wise exp of equation (A4) entails the alternative version of the final size equation in $z = \overline{1} - \sigma$:

$$z = \overline{1} - \exp(-\mathcal{M}z). \tag{A5}$$

Following Andreasen (2011), we interpret equation (A5) as a probabilistic identity as z_i is the probability that agent i becomes infected during the epidemic while $\exp(-\sum_{j\in N} \mathcal{M}_{ij} z_j)$ gives the probability of remaining susceptible during the entire epidemic. The question that remains is whether the final size equation (A4) admits a solution. It is unambiguous to show that the column vector $\sigma^0 = (1, ..., 1)^T$, which corresponds to the disease free equilibrium E_0, yields $\mathcal{H}(\sigma^0) = \overline{0}$, meaning that σ^0 is a solution to the problem (A4). However, more solutions might exist to the final size equation. Using the result

from Andreasen (2011, Theorem 2, p. 2313), we provide the adapted following proposition that specifies conditions for solutions to Eq.(A4).

Proposition 1 *Let $v_1, ..., v_n$ denote the set of eigenvectors and generalized eigenvectors of the next-generation matrix $\mathcal{M}$ and $u_1, ..., u_n$ the set of these vectors squared coordinate-wise. If each u_k is linearly independent of the set of all eigenvectors and generalized eigenvectors excluding v_k, then the final size equation (A4) has a single solution in the open unit $(0,1)^n$ if $R_0 > 1$ and none if $R_0 < 1$.*

Proof To prove Proposition 1, we use bifurcation theory in line with Andreasen (2011) by treating R_0 as a bifurcation parameter. We first show that if $R_0 < 1$, Eq.(A4) admits no solutions in the interior of $(0,1)^n$. Assume the contrary. Then there exists $\sigma \in (0,1)^n$ which solves Eq.(A4). By the Perron-Frobenius Theorem, we can choose the left eigenvector ω corresponding to the dominant eigenvalue of $\mathcal{M}$ (i.e., $\omega^T\ \mathcal{M} = R_0\ \omega^T$) to have positive entries and satisfy $\omega^T\ \overline{1} = 1$. Taking the inner product of ω and Eq.(A4) yields:

$$
\begin{aligned}
0 = \omega^T\ \overline{0} = \omega^T \log\sigma + \omega^T \mathcal{M}(\overline{1} - \sigma) \\
= \omega^T\ \log\sigma + R_0\ \omega^T(\overline{1} - \sigma) \\
\leq \log\ \omega^T\sigma + R_0(1 - \omega^T\sigma) \text{ by Jensen's inequality} \\
\leq (R_0 - 1)(1 - \omega^T\sigma) < 0 \text{ by the inequality } \log y \leq y - 1,
\end{aligned}
$$

which is a contradiction. Therefore if $R_0 < 1$, Eq.(A4) admits no roots in the interior of $(0,1)^n$.

Assume that $R_0 > 1$. Let z^2 and $\log z$ denote the coordinate-wise operations on the vector. Let v_1 be a positive right eigenvector of $\mathcal{M}$ corresponding to R_0 (i.e., $\mathcal{M}v_1 = R_0 v_1$) and $u_1 = v_1^2$. Following Andreasen (2011), we perform an asymptotic expansion of Eq.(A4) in $(R_0 - 1)$ and we show the existence of a feasible root of Eq.(A4) in the form

$$
1 - \sigma = z = (R_0 - 1)\xi_0 v_1 + (R_0 - 1)^2 \sum_k \xi_k v_k + \boldsymbol{O}[(R_0 - 1)^3],
$$

where the coefficients $\xi_0 \neq 0$ and ξ_2 to ξ_n are to be determined. Let ς_k be the subdominant eigenvalues of $\mathcal{M}$. We can rewrite and simplify Eq.(A4) to

$$
\begin{aligned}
0 &= \mathcal{M}z + \log\ (1 - z) \\
&= \mathcal{M}z - z - \frac{1}{2}z^2 + \text{h.o.t.} \\
&= (R_0 - 1)^2\xi_0 v_1 - \frac{1}{2}(R_0 - 1)^2\xi_0^2 u_1 + (R_0 - 1)^2 \sum_k \xi_k(\varsigma_k - 1)v_k + \boldsymbol{O}[(R_0 - 1)^3].
\end{aligned}
$$

Keeping only terms of order $(R_0 - 1)^2$ and letting $\xi'_k = \frac{\xi_k}{\xi_0}$, it follows that ξ_0 and ξ'_k must solve the equation:

$$
v_1 = \frac{1}{2}\xi_0 u_1 + \sum_k \xi'_k(1 - \varsigma_k)v_k.
$$

Assuming that u_1 is linearly independent of the set of subdominant eigenvectors v_2 to v_n ensures the existence and uniqueness of the solution. Since v_1 is independent

of eigenvectors v_2 to v_n, it holds that $\xi_o \neq 0$, and from the reasoning above $\xi_0 > 0$. We have shown that there exists a small, positive solution in z, and consequently of a solution $\sigma = \overline{1} - z \in (0,1)^n$, for $0 < R_0 - 1 \ll 1$.

Note that except for the trivial solution $\sigma^0 = (1, ..., 1)^T$, Eq.(A4) has the same solutions as the vector-equation

$$0 = \mathcal{M}_{kk} + \sum_{j \neq k} \mathcal{M}_{kj} \frac{1-\sigma_j}{1-\sigma_k} + \frac{\log \sigma_k}{1-\sigma_k} = \sum_{j \neq k} \mathcal{M}_{kj} \frac{1-\sigma_j}{1-\sigma_k} + \frac{\log \sigma_k}{1-\sigma_k}, \; k = 1, ..., n, \quad \text{(A6)}$$

since $\mathcal{M}_{kk} = 0$, for $k = 1, .., n$. Considering the left-hand sides of Eq.(A6) as a vector-valued function $G(\sigma_1, ..., \sigma_n)$, and using a similar derivation as in Andreasen (2011, Lemma 3, pp. 2312–2313) , we can show that the determinant of the Jacobian of G is different than zero. The latter means that the number of solutions to $G = 0$ cannot change in the open unit-cube. Therefore, additional solutions to Eq.(A4) cannot arise through bifurcation in the interior of $(0,1)^n$.

Finally, we need to show that Eq.(A4) has no non-trivial solutions on the boundary of $(0,1)^n$. Assume the contrary. Then, there exists $\sigma \neq \sigma^0$ on the boundary of $(0,1)^n$ which solves Eq.(A4). It is direct that $\sigma_k \neq 0$ for all k. Similarly, there are some k for which $\sigma_k = 1$. Since $\log \sigma_k = 0$, from Eq.(A4), the expression on the kth row gives $\sum_l \mathcal{M}_{kl}(1 - \sigma_l) = 0$ implying that $\sigma_l = 1$ for all l. The latter contradicts the fact that $\sigma \neq \sigma^0$.

To conclude the proof, note that we exclude the possibility of additional solutions crossing through the trivial root $\sigma^0 = (1, ..., 1)^T$. In fact, following Andreasen (2011), additional bifurcation at σ^0 may occur only as subdominant eigenvalues pass through unity. However, from the Perron-Frobenious theorem, the associated eigenvectors to the eigenvalues can not be positives on all entries. Using the same reasoning as above to the subdominant eigenvector, we can prove that such solutions cannot enter $(0,1)^n$. Therefore, we conclude the proof. □

Appendix B Theoretical derivations for the planning problem

The current Hamiltonian of problem (2) is given as:

$$\begin{aligned} \mathcal{H}_c(l,x,r,d,s,\mu^1,\mu^2,\mu^3,\mu^4) &= \sum_{i \in N} W_i(k_i,s_i,x_i,r_i,d_i,l_i) + \sum_{i \in N} \mu_i^1 f_i + \gamma \sum_{i \in N} \mu_i^2 x_i \\ &+ \kappa \sum_{i \in N} \mu_i^3 x_i + \sum_{i \in N} \mu_i^4 [-f_i - (\gamma + \kappa) x_i], \end{aligned}$$

where μ_i^j $(j = 1, .., 4)$, for each $i \in N$, are the costate variables. Given the inequality constraints $\dot{x_i} \leq \lambda$, and the constraints $l_i(t) \in [0,1]$, we augment the current Hamiltonian $\mathcal{H}_c$ into the current Lagrangian function:

$$\begin{aligned} \mathcal{L}_c(l,x,r,d,s,\mu^1,\mu^2,\mu^3,\mu^4,\theta^1,\theta^2,\theta^3) &= \sum_{i \in N} W_i(k_i,s_i,x_i,r_i,d_i,l_i) + \sum_{i \in N} \mu_i^1 f_i \\ &+ \gamma \sum_{i \in N} \mu_i^2 x_i + \kappa \sum_{i \in N} \mu_i^3 x_i + \sum_{i \in N} \mu_i^4 [-f_i - (\gamma + \kappa) x_i] \\ &+ \sum_{i \in N} \theta_i^1 (\lambda - f_i) + \sum_{i \in N} \theta_i^2 l_i + \sum_{i \in N} \theta_i^3 (1 - l_i), \end{aligned}$$

where the parameters θ^j, $j = 1, 2, 3$, are Lagrange multipliers. We can also rewrite $\mathcal{L}_c$ as:

$$\begin{aligned}\mathcal{L}_c(l, x, r, d, s, \mu^1, \mu^2, \mu^3, \mu^4, \theta^1, \theta^2, \theta^3) = & \sum_{i \in N} W_i(k_i, s_i, x_i, r_i, d_i, l_i) + \sum_{i \in N} (\mu_i^1 - \mu_i^4 - \theta_i^1) f_i \\ & + \gamma \sum_{i \in N} \mu_i^2 x_i + \kappa \sum_{i \in N} \mu_i^3 x_i - (\gamma + \kappa) \sum_{i \in N} \mu_i^4 x_i \\ & + \lambda \sum_{i \in N} \theta_i^1 + \sum_{i \in N} \theta_i^2 l_i + \sum_{i \in N} \theta_i^3 (1 - l_i).\end{aligned}$$

The first-order conditions for maximizing $\mathcal{L}_c$ call for, assuming interior solutions,

$$\frac{\partial \mathcal{L}_c}{\partial l_k} = 0, \ k \in N, \tag{B7}$$

as well as for each $k \in N$:

$$\frac{\partial \mathcal{L}_c}{\partial \theta_k^1} = \lambda - \dot{x_k} \geq 0, \qquad \theta_k^1 \geq 0, \qquad \theta_k^1 \frac{\partial \mathcal{L}_c}{\partial \theta_k^1} = \theta_k^1 (\lambda - \dot{x_k}) = 0, \tag{B8}$$

$$\frac{\partial \mathcal{L}_c}{\partial \theta_k^2} = l_k \geq 0, \qquad \theta_k^2 \geq 0, \qquad \theta_k^2 \frac{\partial \mathcal{L}_c}{\partial \theta_k^2} = \theta_k^2 l_k = 0, \text{ and} \tag{B9}$$

$$\frac{\partial \mathcal{L}_c}{\partial \theta_k^3} = 1 - l_k \geq 0, \qquad \theta_k^3 \geq 0, \qquad \theta_k^3 \frac{\partial \mathcal{L}_c}{\partial \theta_k^3} = \theta_k^3 (1 - l_k) = 0. \tag{B10}$$

Finally, the other maximum-principle conditions that include the dynamics for state and co-state variables are, for $k \in N$:

$$\dot{x_k} = \frac{\partial \mathcal{L}_c}{\partial \mu_k^1} \qquad \dot{r_k} = \frac{\partial \mathcal{L}_c}{\partial \mu_k^2} \qquad \dot{d_k} = \frac{\partial \mathcal{L}}{\partial \mu_k^3} \qquad \dot{s_k} = \frac{\partial \mathcal{L}_c}{\partial \mu_k^4}, \text{ and}$$

$$\dot{\mu_k^1} = \delta \mu_k^1 - \frac{\partial \mathcal{L}_c}{\partial x_k} \quad \dot{\mu_k^2} = \delta \mu_k^2 - \frac{\partial \mathcal{L}_c}{\partial r_k} \quad \dot{\mu_k^3} = \delta \mu_k^3 - \frac{\partial \mathcal{L}_c}{\partial d_k} \quad \dot{\mu_k^4} = \delta \mu_k^4 - \frac{\partial \mathcal{L}_c}{\partial s_k} \tag{B11}$$

Recall that $f_i(x_i, r_i, d_i, l_i) = \beta(1 - x_i - r_i - d_i)(1 - l_i) \sum_{j \neq i} [A_{ij}(1 - l_j) x_j] - (\gamma + \kappa) x_i$.

Then,

$$\frac{\partial f_i}{\partial l_k} = \begin{cases} -\beta(1 - x_i - r_i - d_i) \sum_{j \neq i} [A_{ij}(1 - l_j) x_j] & \text{if } k = i \\ -\beta(1 - x_i - r_i - d_i)(1 - l_i) A_{ik} x_k & \text{if } k \neq i \end{cases}$$

$$\frac{\partial f_i}{\partial x_k} = \begin{cases} -\beta(1 - l_i) \sum_{j \neq i} [A_{ij}(1 - l_j) x_j] - (\gamma + \kappa) & \text{if } k = i \\ \beta(1 - x_i - r_i - d_i)(1 - l_i)(1 - l_k) A_{ik} & \text{if } k \neq i \end{cases}$$

$$\frac{\partial f_i}{\partial r_k} = \frac{\partial f_i}{\partial d_k} = \begin{cases} -\beta(1 - l_i) \sum_{j \neq i} [A_{ij}(1 - l_j) x_j] & \text{if } k = i \\ 0 & \text{if } k \neq i \end{cases}$$

We also recall that $W_i(k_i, s_i, x_i, r_i, d_i, l_i) = p_i y_i(k_i, s_i, x_i, r_i, d_i, l_i) - w_i h_i(s_i, x_i, r_i, d_i, l_i)$. Therefore, for each i and k, and for each $u \in \{s_k, x_k, r_k, d_k, l_k\}$, it holds that

$$\frac{\partial W_i}{\partial u} = \begin{cases} p_i \frac{\partial y_i}{\partial u} - w_i \frac{\partial h_i}{\partial u} & \text{if } k = i \\ 0 & \text{if } k \neq i \end{cases} \tag{B12}$$

Therefore, for each $k \in N$, we can write $\frac{\partial \mathcal{L}_c}{\partial l_k}$ as:

$$\begin{aligned}\frac{\partial \mathcal{L}_c}{\partial l_k} &= \sum_{i\in N}\frac{\partial W_i}{\partial l_k} + \sum_{i\in N}(\mu_i^1 - \mu_i^4 - \theta_i^1)\frac{\partial f_i}{\partial l_k} + \theta_k^2 - \theta_k^3 \\ &= \frac{\partial W_k}{\partial l_k} + \sum_{i\in N}(\mu_i^1 - \mu_i^4 - \theta_i^1)\frac{\partial f_i}{\partial l_k} + \theta_k^2 - \theta_k^3 \text{ using } (B12) \\ &= p_k\frac{\partial y_k}{\partial l_k} - w_k\frac{\partial h_k}{\partial l_k} + \sum_{i\in N}(\mu_i^1 - \mu_i^4 - \theta_i^1)\frac{\partial f_i}{\partial l_k} + \theta_k^2 - \theta_k^3\end{aligned} \tag{B13}$$

Hence, using the first-order conditions (B7), equation (B13) becomes:

$$0 = p_k\frac{\partial y_k}{\partial l_k} - w_k\frac{\partial h_k}{\partial l_k} + \sum_{i\in N}(\mu_i^1 - \mu_i^4 - \theta_i^1)\frac{\partial f_i}{\partial l_k} + \theta_k^2 - \theta_k^3.$$

Using the other conditions from (B11) and using (B12):

$$\dot{\mu}_k^1 = \delta\mu_k^1 - \frac{\partial \mathcal{L}_c}{\partial x_k} = \delta\mu_k^1 - p\frac{\partial y_k}{\partial x_k} + w_k\frac{\partial h_k}{\partial x_k} - \gamma\mu_k^2 - \kappa\mu_k^3 + (\gamma+\kappa)\mu_k^4 - \sum_{i\in N}(\mu_i^1 - \mu_i^4 - \theta_i^1)\frac{\partial f_i}{\partial x_k}. \tag{B14}$$

Similarly, using (B11), we get:

$$\dot{\mu}_k^2 = \delta\mu_k^2 - \frac{\partial \mathcal{L}_c}{\partial r_k} = \delta\mu_k^2 - p\frac{\partial y_k}{\partial r_k} + w_k\frac{\partial h_k}{\partial r_k} - \sum_{i\in N}(\mu_i^1 - \mu_i^4 - \theta_i^1)\frac{\partial f_i}{\partial r_k} \text{ using } (B12), \tag{B15}$$

$$\dot{\mu}_k^3 = \delta\mu_k^3 - \frac{\partial \mathcal{L}_c}{\partial d_k} = \delta\mu_k^3 - p\frac{\partial y_k}{\partial d_k} + w_k\frac{\partial h_k}{\partial d_k} - \sum_{i\in N}(\mu_i^1 - \mu_i^4 - \theta_i^1)\frac{\partial f_i}{\partial d_k} \text{ using } (B12), \tag{B16}$$

and

$$\dot{\mu}_k^4 = \delta\mu_k^4 - \frac{\partial \mathcal{L}_c}{\partial s_k} = \delta\mu_k^4 - p\frac{\partial y_k}{\partial s_k} + w_k\frac{\partial h_k}{\partial s_k}. \tag{B17}$$

Appendix C Proofs of Theoretical Results

C.1 Proof of Proposition 1

Given that $s_i = 1 - x_i - r_i - d_i$, for each $i \in N$, we can rewrite (ODE) as:

$$(\text{ODE}) : \begin{cases} \dot{x_i} = \beta(1 - x_i - r_i - d_i)(1 - l_i)\sum_{j\in N}[A_{ij}(1 - l_j)x_j] - (\gamma + \kappa)x_i \\ \dot{s_i} = -\beta(1 - x_i - r_i - d_i)(1 - l_i)\sum_{j\in N}[A_{ij}(1 - l_j)x_j] \\ \dot{r_i} = \gamma x_i \\ \dot{d_i} = \kappa x_i \end{cases}$$

Consider the vector-valued function $f_i(t, X_i) = (f_{i1}(t, X_i), f_{i2}(t, X_i), f_{i3}(t, X_i), f_{i4}(t, X_i))^T$, where

$$f_{i1}(t, X_i) = \beta(1 - x_i - r_i - d_i)(1 - l_i)\sum_{j\in N}[A_{ij}(1 - l_j)x_j] - (\gamma + \kappa)x_i$$

$$f_{i2}(t, X_i) = -\beta(1 - x_i - r_i - d_i)(1 - l_i)\sum_{j\in N}[A_{ij}(1 - l_j)x_j]$$

$$f_{i3}(t, X_i) = \gamma x_i \text{ and}$$
$$f_{i4}(t, X_i) = \kappa x_i.$$

The function f_i is a continuously differentiable function, for each $i \in N$. Consequently, the ODE admits a unique solution, $\mathcal{S}^*(l, A, \beta, \gamma, \kappa, X_0)$, thanks to the theorem of existence and uniqueness of a solution for first-order general ordinary differential equations, where $l = (l_i)_{i \in N} \in [0, 1]^n$ is a vector of individual lockdown probabilities.

C.2 Proof of Proposition 2

Let $\mathcal{J} = \mathcal{A} - \mathcal{B}$. We denote, $s(\mathcal{J})$, the maximum real part of all the eigenvalues of the matrix $\mathcal{J}$ (the spectral abscissa of $\mathcal{J}$). Following Van den Driessche and Watmough (2002, Lemma 1, p. 32), the DFE E_0 of system (ODE) is locally-asymptotically stable if all the eigenvalues of the matrix $\mathcal{J}$ have negative real parts (i.e., $s(\mathcal{J}) < 0$), and unstable if any eigenvalue of $\mathcal{J}$ has a positive real part (i.e., $s(\mathcal{J}) > 0$). A matrix $A = [A_{ij}]$ has the Z-sign pattern if $A_{ij} \leq 0$ for all $i \neq j$. It is direct that the matrix $\mathcal{B}$ has the Z-sign pattern. The eigenvalues of $\mathcal{B}$ have positive real parts. Thus, $\mathcal{B}$ is a non-singular M-matrix (for additional information on M-matrices, we refer to Van den Driessche and Watmough (2002) and the references therein). Also, the matrix $-\mathcal{J}$ have the Z-sign pattern. Thus, $s(\mathcal{J}) < 0$ if and only if $-\mathcal{J}$ is a non-singular M-matrix. Since $\mathcal{M}$ is non-negative, then $-\mathcal{J}\mathcal{B}^{-1} = I - \mathcal{M}$ also has the Z-sign pattern. Applying Van den Driessche and Watmough (2002, Lemma 5, p. 47), $-\mathcal{J}$ is a non-singular M-matrix if and only if $I - \mathcal{M}$ is a non-singular M-matrix. Since the matrix $\mathcal{M}$ is non-negative, all the eigenvalues of $\mathcal{M}$ have magnitude less than or equal to $\rho(\mathcal{M})$. Thus, $I - \mathcal{M}$ is a non-singular M-matrix if and only if $\rho(\mathcal{M}) < 1$. Hence, $s(\mathcal{J}) < 0$ if and only if $R_0 = \rho(\mathcal{M}) < 1$. Similarly, it follows that $s(\mathcal{J}) = 0$ if and only if $-\mathcal{J}$ is a singular M-matrix. The latter is equivalent to $I - \mathcal{M}$ is a singular M-matrix (applying Van den Driessche and Watmough (2002, Lemma 6, p. 47)). Thus, $I - \mathcal{M}$ is a singular M-matrix if and only $\rho(\mathcal{M}) = 1$. Consequently, $s(\mathcal{J}) = 0$ if and only if $\rho(\mathcal{M}) = 1$. Therefore, it follows that $s(\mathcal{J}) > 0$ if and only if $R_0 = \rho(\mathcal{M}) > 1$.

C.3 Proof of Proposition 3

We denote $f_i(k_i, s_i, x_i, r_i, d_i, l_i) \equiv f_i(k_i, x_i, r_i, d_i, l_i) = \beta[1 - x_i - r_i - d_i](1 - l_i) \sum_{j \in N} [A_{ij}(1 - l_j)x_j] - (\gamma + \kappa)x_i$, and $W_i(k_i, s_i, x_i, r_i, d_i, l_i) = p_i y_i(k_i, s_i, x_i, r_i, d_i, l_i) - w_i h_i(s_i, x_i, r_i, d_i, l_i)$. The function $l_i : t \longrightarrow l_i(t) \in [0, 1]$ is continuous. The function W_i, and the objective function in (2) are continuous and differentiable. Moreover, f_i and the right-hand sides of the laws of motion in (2) are all continuous and differentiable. It follows that the problem (2) admits a unique optimal path $\{l^*(t)\}$ of the control variable (and the states $\{x^*(t), r^*(t), d^*(t), s^*(t)\}$, given the initial conditions X_0 and the laws of motion).

Appendix D Optimal Dynamics with Simulations

We obtain our optimal lockdown dynamics by solving the planning problem described in Eq.(2). The simulation process involves solving Eqs.(B7) to (B11), and (B14) to

(B17). The lockdown dynamics yield the disease and economic costs dynamics. We use the software **Matlab_2020a** and the function **ode45** to solve the system of ordinary differential equations describing the N-SIRD epidemiological model. The existence and uniqueness of the solution for our epidemiological model and planning problem are established in Propositions 1 and 3, respectively. These conditions ensure that the output from our simulations is the appropriate approximation of the N-SIRD optimal dynamics. As initial inputs in the simulations, we specify the adjacency matrix A, representing the social network structure, and randomly impose 10% of infected agents. At the initial period, there is no agent in lockdown. For each period $t \in \{1, 2, ..., 80\}$, the simulation program optimally produces individual probabilities for each of our variables of interest. Then, we represent the average probability in the sample for each point in time t in Figs. 1, 3, and 4. We can view these dynamics as representative of the population dynamics and thus useful for policy analysis and decision making.

Appendix E Definitions of Network Metrics

We recall that the network A is a symmetric $n \times n$ weighted adjacent matrix (A_{ij}). An agent's *degree centrality* χ_i equals the total number of other agents directly connected to agent i (i.e., the number of agent i's neighbors): $\chi_i = \sum_{j=1}^{n} A_{ij}$. *Eigenvector centrality* ν_i measures the extent to which agent i is connected to other highly connected agents in the network A: $\nu_i = \frac{1}{e} \sum_{j=1}^{n} A_{ij}\nu_j$. The eigenvector centrality is computed using the principal eigenvector ν of the adjacent matrix A, that we can write in matrix notation as $A\nu = e\nu$, where ν is a column vector with n entries. The eigenvector centrality reflects the notion that connections to highly connected agents are more important. Agent i's betweenness centrality, b_i, measures the fraction of shortest paths passing through agent i: $b_i = \sum_{j,k} \frac{\sigma_{jk}^i}{\sigma_{jk}}$, where σ_{jk} is the total number of shortest paths from agents j to k, and σ_{jk}^i is the number of those paths that pass through agent i. Agent i's *closeness centrality*, c_i, measures how close is the agent to all other agents in the network A: $c_i = \frac{1}{\sum_j d(i,j)}$, where $d(i, j)$ is the distance (or shortest path) between agents i and j. It follows from these definitions that the degree centrality is less based on network configuration than the other centrality measures.

Appendix F Estimating Tolerable Infection Incidence: Additional Details

F.1 Calibrate a U.S. nursing home production function

In our study, we use data from several sources. Our primary source of external data comes from the Protect Nursing Homes project and the replication package made publicly available by Chen, Chevalier, and Long (2021). Table 2 describes the relevant parameters, their sources, and their use in our simulations and empirical analyses. For calibration, we assume that the production structure is homogeneous within a U.S. state. To estimate the production function for a nursing home i in a U.S. state, we assume a Cobb-Douglas function $y_i = k_i^{\alpha^1} h_i^{\alpha^2}$, where y_i is the total

number of residents who receive care (output), k_i is the total number of beds (proxy for capital), and h_i is the number of occupied beds (a proxy for the labor demand). We assume that a nursing home hires staff according to the demand for its services, and the latter is closely related to the number of occupied beds. We estimate the elasticity α^1 and α^2 by assuming a log-log specification and controlling for a series of factors, including overall rating, County SSA, CMS quality rating, urban/rural, and for-profit/not-for-profit. We use the following simple log-log econometric equation:

$$log(y_i) = \alpha^0 + \alpha^1 log(k_i) + \alpha^2 log(h_i) + \beta X_i + e_i,$$

where X_i represents exogenous nursing home i's characteristics and e_i is the error term. The estimates of the parameter α^1 ($\alpha^1 = \alpha$, $\alpha^2 \approx 1-\alpha$) for each U.S. state and other state's parameters that we use to simulate the N-SIRD model are presented in Table F1.

Springer Nature 2021 LaTeX template

F.2 Estimation of the Tolerable Infection Incidence Parameters

Our study uses the simulated minimum distance estimator to estimate the tolerable COVID-19 infection incidence value for all U.S. states in our sample. Given the lack of data on daily COVID-19 deaths in the nursing home data provided by Chen et al. (2021), we use the New York Times' daily COVID-19 death count for each U.S. state between May 31 and August 16, 2020. Following National Center for Health Statistics (2020), Freed, Cubanski, Neuman, Kates, and Michaud (2020), and Powell, Bellin, and Ehrlich (2020), we assume that 80% of U.S. COVID-19 deaths on average are seniors (65 years and older). We use these figures as our observed outcome.

Let us index a U.S. state in Chen et al. (2021)'s data set by $s \in \overline{S}$, with $\overline{S} = \{1, ..., 49\}$. Let d_{ts} denote the number of COVID-19 deaths observed at time $t = 1, ..., \bar{t}$ in the U.S. state $s \in \overline{S}$. For each value of the tolerable infection incidence λ, we can simulate death dynamics denoted as $\hat{d}_{ts}(\lambda)$. Since our simulations are deterministic, there is no random shock in our model. Thus, repeating the simulations with the same initial conditions produce the same results. For each U.S. state, we estimate the parameter λ that we denote as $\hat{\lambda}_s$ by solving the following minimization problem:

$$\hat{\lambda}_s = argmin \left\{ \sum_{t=1}^{\bar{t}} (\hat{d}_{ts}(\lambda) - d_{ts})^2 \right\}, \ \lambda \in [0, 1]. \tag{F18}$$

Existing literature on simulated minimum distance estimators (e.g., Gertler and Waldman (1992), and Forneron and Ng (2018)) suggests that $\hat{\lambda}_s$ is a consistent estimator of the tolerable COVID-19 infection incidence level for each U.S. state. The simulated minimum distance produces $\hat{\lambda}_s$ for 26 U.S. states. For the remaining 23 U.S. states, the parameter λ is not identified. This group of states can be divided into two. The first group consists of three states, NC, UT, and VA. For these U.S. states, the available epidemiological parameters and the data of their nursing home networks do not support our model. This usually occurs when the next-generation matrix, $\mathcal{M}$, cannot be obtained. We can refer to Proposition 2, which studies the asymptotic stability of the system (ODE) at the disease-free equilibrium E_0, and provides conditions for which an infection in a fully susceptible population could generate an epidemic outbreak. The proof of Proposition 2 in Appendix C.2 transforms such conditions into properties (e.g., non-singularity) placed on the next-generation matrix, $\mathcal{M}$, which also depends on the network structure of nursing homes in the U.S. state. For the second group which consists of the remaining 20 U.S. states, it is impossible for us to exclude zero tolerable infection tolerance (i.e., $\lambda = 0$) or any other level $\lambda \in]0, 1]$. Indeed, the estimation procedure always returns the initial value proposed, suggesting a flat objective function. We conjecture that the main reason that may justify this outcome is data quality. Indeed, the raw daily COVID-19 death count data from the New York Times is not specifically collected for the residents of nursing homes or for the population of seniors in the state. Because of data availability issues, and following National Center for Health Statistics (2020), Freed et al. (2020), and Powell et al. (2020), we assume that approximately 80% of daily U.S. COVID-19 deaths come from nursing homes. The death dynamics will match in U.S. states where most seniors are in nursing homes and their daily death counts follow the same trend as the state's COVID-19 death count. This means that in practice, there will be a potential mismatch between the simulation-based dynamic and the raw data dynamic.

Table F1: Data used to simulate the N-SIRD model for each U.S. state

U.S. States	Wage/hour	Price/hour	Case/100000	Death/100000	R_0	α^1	Min. Degree
AL	33	9.29	365.9	59.3	0.93	0.3853637	13
AR	43	11.04	281.4	59.6	0.95	0.5218234	13
AZ	35	8.79	241.2	45.8	0.97	0.5852448	13
CA	52	15.37	274.8	51.8	0.86	0.6297461	13
CO	42	13.08	109.6	38.4	0.92	0.538735	13
CT	46	18.81	345	100	0.96	0.6257474	13
DC	42	17.07	229.9	56.3	0.92	1.002931	1
DE	52	19.30	188.3	55.3	0.97	0.7739902	1
FL	39	13.19	283	53.1	0.97	0.6614283	22
GA	38	9.64	356.1	73.7	0.84	0.4615252	22
IA	38	12.26	131.2	31	0.92	0.2876858	1
ID	40	9.44	162	22.8	0.87	0.2217902	1
IL	39	11.41	209	52.8	0.87	0.4060938	11
IN	36	9.61	159.8	59	0.88	0.2871411	11
KS	35	9.44	83.6	16	0.96	0.3383333	11
KY	34	10.67	162.4	34.9	1.01	0.331612	11
LA	34	7.85	418.6	85.5	0.89	0.2518069	11
MA	42	14.27	356.1	125.1	0.94	0.76337	11
MD	45	14.31	263.1	62.4	0.94	0.6189594	11
ME	47	18.19	48.3	9	0.96	0.7124791	1
MI	39	13.08	138.2	48.8	0.9	0.6715142	9
MN	44	16.16	100.9	33.8	0.94	0.5657483	9
MO	32	9.61	180.6	30.8	0.94	0.2763414	9
MS	36	7.73	367	74.9	1.08	0.6377482	1
MT	41	11.12	23.5	4.7	0.87	0.0505241	1
NC	38	10.66	221.9	46.1	0.92	0.5917483	NaN
ND	45	14.23	102.3	15	1.09	0.4858396	2
NE	45	15.21	94.2	26.4	0.94	0.3416526	2
NH	47	15.94	141.9	38.3	0.95	0.5850638	2
NJ	42	11.16	365.8	120.1	1.03	0.7126257	2
NM	46	17.38	138.6	50.1	0.84	0.549336	2
NV	38	10.83	209.9	26.4	0.95	0.7162963	2
NY	38	17.16	145.2	50.5	0.91	0.7638234	2
OH	38	11.04	149.2	34.9	0.88	0.5926255	2
OK	36	7.77	148.2	22.2	1.05	0.4150358	2
OR	48	15.02	74.5	13.6	0.94	0.2144981	2
PA	39	14.55	207.1	66.2	0.88	0.6404236	9
RI	46	13.74	288.8	86.6	0.92	0.9319015	9
SC	37	10.57	342.2	63.1	0.9	0.5003951	9
SD	35	10.11	78.9	14.4	0.94	0.3584688	1
TN	34	10.24	166.5	24.8	0.87	0.2476998	9
TX	38	8.59	325.7	62	0.97	0.3152237	22
UT	37	11.24	134	31.9	0.98	0.3648363	NaN
VA	41	13.86	192.3	46.2	1.05	0.6377195	NaN
VT	41	11.86	35	11.1	1.11	0.6885093	2
WA	47	14.72	152.7	38	0.97	0.6368927	9
WI	35	16.31	64	12.4	0.91	0.2780716	9
WV	41	12.67	101.7	17.8	0.87	0.2662697	9
WY	42	11.71	8.4	2.2	0.99	0.2333506	1

Note: Min. Degree is the minimum degree centrality of the nursing homes kept in the sample to ensure the the next-generation matrix, $\mathcal{M}$ can be computed. “NaN” in the table means “Non Available.” For these three U.S. states, there was no degree centrality level for which we can simulate the N-SIRD model with the available epidemiological and economic parameters.

Appendix G Robustness Checks

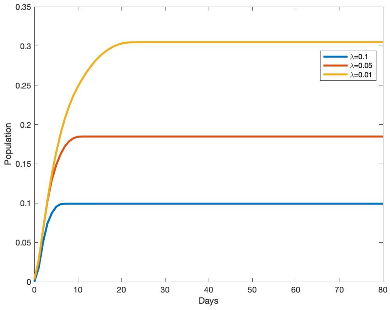

(a) Dynamics of lockdown

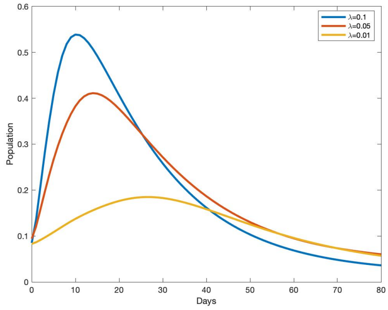

(b) Dynamics of infection

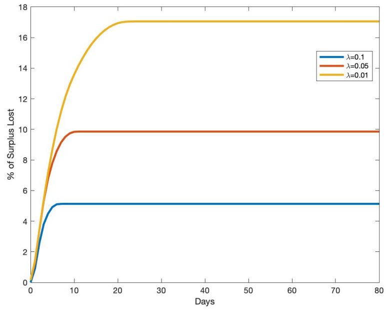

(c) Dynamics of economic Cost

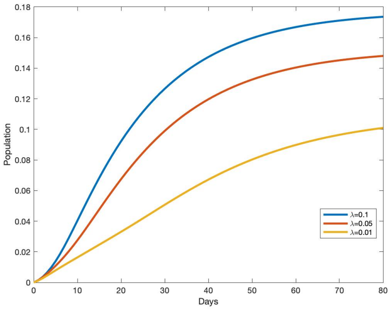

(d) Dynamics of death

Fig. G1: Health versus wealth tradeoff in a scale-free network. Note: We perform three sets of simulations with three different values of the tolerable infection incidence λ: 0.01, 0.05, and 0.1. The results are displayed in a two-dimensional graphic, with days on the horizontal axis, and the percentage of population affected for the variable (infection, lockdown, or death) illustrated on the vertical axis. In each period, a point in the graphic represents the average value of individual probabilities. For the economic cost, the vertical axis represents the percentage of economic surplus lost relative to the economy without the pandemic. Each graph shows three curves corresponding to three dynamics for a single variable of interest for a given value of λ. All variability within each curve in each graph is a result of the stochastic nature of transmission and not variation in the network or λ.

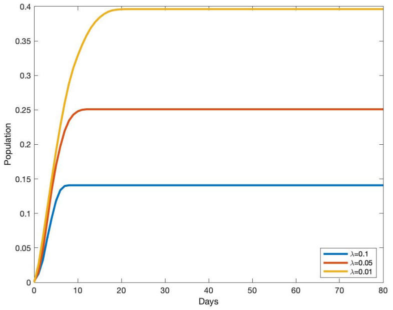

(a) Dynamics of lockdown

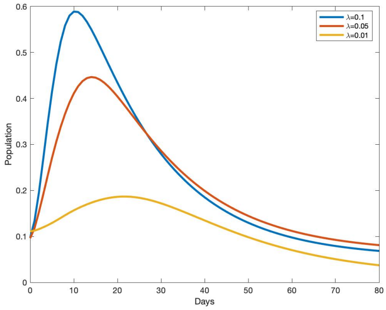

(b) Dynamics of infection

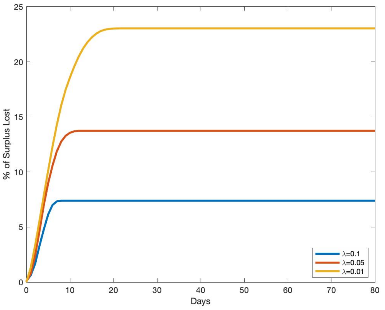

(c) Dynamics of economic Cost

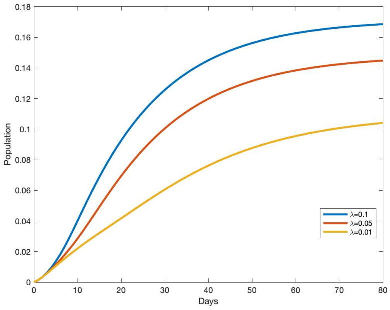

(d) Dynamics of death

Fig. G2: Health versus wealth tradeoff in a random network. Note: We perform three sets of simulations with three different values of the tolerable infection incidence λ: 0.01, 0.05, and 0.1. The results are displayed in a two-dimensional graphic, with days in the horizontal axis, and the percentage of population affected for the variable (infection, lockdown, or death) illustrated on the vertical axis. In each period, a point in the graphic represents the average value of individual probabilities. For the economic cost, the vertical axis represents the percentage of economic surplus lost relative to the economy without the pandemic. Each graph shows three curves corresponding to three dynamics for a single variable of interest for a given value of λ. All variability within each curve in each graph is a result of the stochastic nature of transmission and not variation in the network or λ.

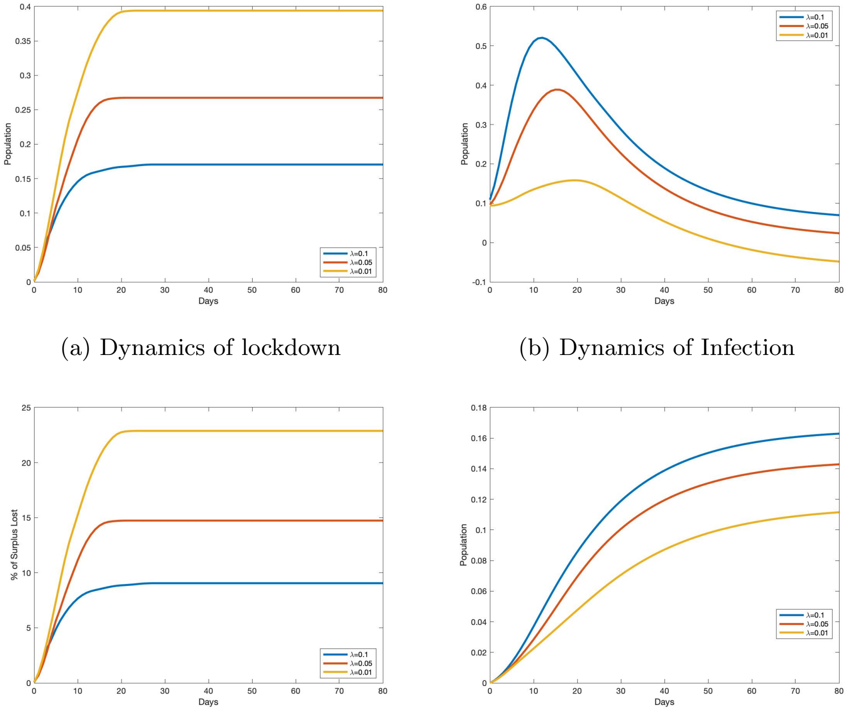

(a) Dynamics of lockdown

(b) Dynamics of Infection

(c) Dynamics of Economic Cost

(d) Dynamics of Death

Fig. G3: Health versus wealth tradeoff in a lattice network. Note: We perform three sets of simulations with three different values of the tolerable infection incidence λ: 0.01, 0.05, and 0.1. The results are displayed in a two-dimensional graphic, with days in the horizontal axis, and the percentage of population affected for the variable (infection, lockdown, or death) illustrated on the vertical axis. In each period, a point in the graphic represents the average value of individual probabilities. For the economic cost, the vertical axis represents the percentage of economic surplus lost relative to the economy without the pandemic. Each graph shows three curves corresponding to three dynamics for a single variable of interest for a given value of λ. All variability within each curve in each graph is a result of the stochastic nature of transmission and not variation in the network or λ.

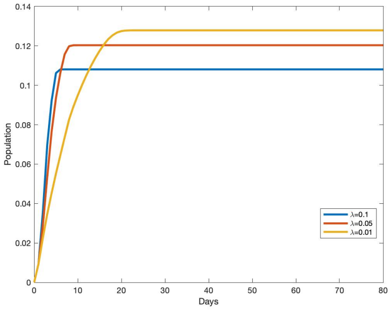

(a) Dynamics of Lockdown

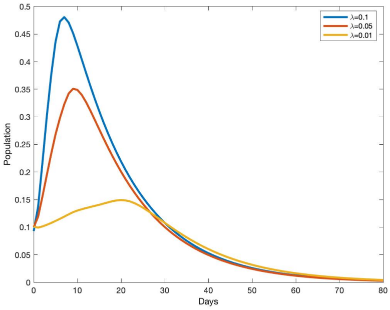

(b) Dynamics of Infection

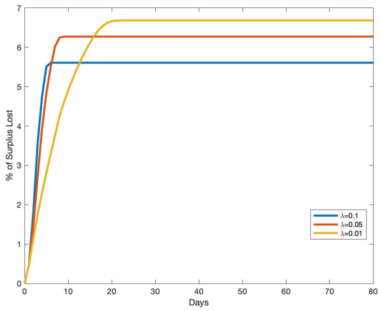

(c) Dynamics of Economic Cost

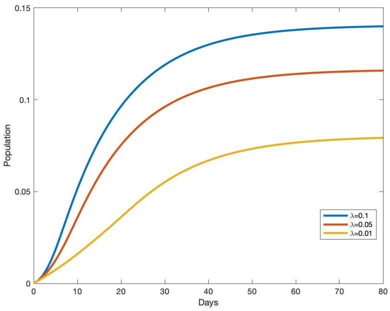

(d) Dynamics of Death

Fig. G4: Health versus wealth tradeoff with COVID-19 Delta variant parameters in a small-world network. Note: We perform three sets of simulations with three different values of the tolerable infection incidence λ: 0.01, 0.05, and 0.1. The epidemiological parameters are set to match the transmissibility of the COVID-19 Delta variant ($R_0 = 5.08$; see Liu and Rocklöv (2021)). The infectious period is set to 14 days and the recovery and death rates are left unchanged. The results are displayed in a two-dimensional graphic, with days on the horizontal axis, and the percentage of population affected for the variable (infection, lockdown, or death) on the vertical axis. In each period, a point in the graphic represents the average value of individual probabilities. For the economic cost, the vertical axis represents the percentage of economic surplus lost relative to the economy without the pandemic. Each graph shows three curves corresponding to three dynamics for a single variable of interest for a given value of λ. All variability within each curve in each graph is a result of the stochastic nature of transmission and not variation in the network or λ.

Table G2: Network centrality and lockdown probability in lattice, random, and scale-free network configurations

		$\lambda = 0.1$			$\lambda = 0.05$			$\lambda = 0.01$		
		Lattice	Random	Scale Free	Lattice	Random	Scale Free	Lattice	Random	Scale Free
Eigenvalue	corr	-2.09e-16	0.1988	0.2733	-1.39e-15	0.2049	0.0203	-1.15e-15	0.2045	0.015
	p-value	1	2.26e-10	1.35e-18	1	6.17e-11	0.5223	1	6.70e-11	0.6362
Degree	corr	NaN	0.2339	0.4105	NaN	0.2389	0.1239	NaN	0.2731	0.1302
	p-value	NaN	6.81e-14	6.23e-42	NaN	1.91e-14	8.55e-05	NaN	1.47e-18	3.62e-05
Betweenness	corr	0.0034	0.2167	0.2647	6.31e-04	0.2116	0.0437	0.0015	0.2348	0.0434
	p-value	0.915	4.32e-12	1.72e-17	0.9841	1.37e-11	0.1675	0.9617	5.39e-14	0.1701
Closeness	corr	2.03e-16	0.2202	0.4006	1.39e-15	0.2426	0.0954	1.15e-15	0.2509	0.0591
	p-value	1	1.91e-12	7.83e-40	1	7.35e-15	0.0025	1	7.99e-16	0.0618

Note: The p-value for each centrality measure is for the test of the hypothesis H_0 $\rho = 0$ vs H_1 $\rho \neq 0$. The label "NaN" means "Non available". In the lattice network, all individuals have the same degree. Thus, we cannot compute the correlation between the degree centrality and the optimal lockdown probability. For the random network, we observe a strong correlation between each centrality measure and the optimal lockdown probability. The lockdown dynamics and correlations in the scale-free and small-world networks move in the same direction.

Table G3: Network centrality and lockdown probability in a small-world network with COVID-19 Delta variant parameters

λ	Eigenvalue		Degree		Betweenes		Closeness	
	corr	p-value	corr	p-value	corr	p-value	corr	p-value
0.1	0.2121	1.24e-11	0.2769	4.67e-19	0.231	1.39e-13	0.2277	3.17e-13
0.05	0.2055	5.38e-11	0.2696	4.04e-18	0.2296	2.00e-13	0.2386	2.09e-14
0.01	0.1012	0.0014	0.1829	5.66e-09	0.1334	2.30e-05	0.1513	1.53e-06

Note: Table G3 illustrates the correlation (corr) between measures of centrality and average optimal lockdown probability in a small-world network with COVID-19 Delta variant parameters for three values of λ. The p-value for each centrality measure is for the test of the hypothesis H_0 $\rho = 0$ vs H_1 $\rho \neq 0$.

Table G4: Estimation of laissez-faire policies in U.S. nursing homes with degree centrality

	(1)	(2)	(3)	(4)	(5)
λ	0.197	1.129**	1.700**	-0.451	1.601**
	(0.57)	(3.02)	(2.58)	(-0.89)	(2.19)
Degree Centrality	0.0616***	0.0900***	0.0622***	0.0612***	0.0903***
	(6.34)	(6.79)	(6.41)	(6.29)	(6.75)
County_ssa	-0.000773	-0.000824	-0.000501	-0.000786	-0.000598
	(-1.09)	(-1.17)	(-0.70)	(-1.11)	(-0.84)
D_Profit	0.208*	0.211*	0.210*	0.0772	0.0358
	(1.76)	(1.79)	(1.78)	(0.56)	(0.26)
$\lambda\times$ Degree Centrality		-0.169***			-0.170***
		(-3.12)			(-3.07)
$\lambda\times$ County_ssa			-0.00475***		-0.00424**
			(-2.64)		(-2.39)
$\lambda\times$ D_Profit				0.980	1.327**
				(1.57)	(2.03)
Overall_rating	-0.188***	-0.182***	-0.188***	-0.191***	-0.185***
	(-4.70)	(-4.52)	(-4.68)	(-4.75)	(-4.58)
County FE	Yes	Yes	Yes	Yes	Yes
Observations	6478	6478	6478	6478	6478
R^2	0.078	0.079	0.079	0.078	0.080

Note: The dependant variable is the total number of COVID-19 deaths in the nursing Home. t statistics in parentheses, * $p < 0.10$, ** $p < 0.05$, *** $p < 0.01$. Standard errors are robust to heteroscedasticity of unknown form.

References

Andreasen, V. (2011). The final size of an epidemic and its relation to the basic reproduction number. *Bulletin of Mathematical Biology*, *73*(10), 2305–2321.

Brauer, F. (2008). Epidemic models with heterogeneous mixing and treatment. *Bulletin of Mathematical Biology*, *70*(7), 1869.

Chen, M.K., Chevalier, J.A., Long, E.F. (2021). Nursing home staff networks and COVID-19. *Proceedings of the National Academy of Sciences*, *118*(1).

Forneron, J.-J., & Ng, S. (2018). The ABC of simulation estimation with auxiliary statistics. *Journal of Econometrics*, *205*(1), 112–139.

Freed, M., Cubanski, J., Neuman, T., Kates, J., Michaud, J. (2020). *What share of people who have died of COVID-19 are 65 and older – and how does it vary by state?* https://www.kff.org/coronavirus-covid-19/issue-brief/what-share-of-people-who-have-died-of-covid-19-are-65-and-older-and-how-does-it-vary-by-state/. (Accessed: 2021-10-12)

Gertler, P.J., & Waldman, D.M. (1992). Quality-adjusted cost functions and policy evaluation in the nursing home industry. *Journal of Political Economy*, *100*(6), 1232–1256.

Liu, Y., & Rocklöv, J. (2021). The reproductive number of the delta variant of SARS-CoV-2 is far higher compared to the ancestral SARS-CoV-2 virus. *Journal of Travel Medicine*, *28*(7), 1–3.

National Center for Health Statistics (2020). *Provisional COVID-19 deaths by sex and age.* https://data.cdc.gov/NCHS/Provisional-COVID-19-Deaths-by-Sex-and-Age/9bhg-hcku. (Accessed: 2021-10-12)

Powell, T., Bellin, E., Ehrlich, A.R. (2020). Older adults and COVID-19: the most vulnerable, the hardest hit. *Hastings Center Report*, *50*(3), 61–63.

Van den Driessche, P., & Watmough, J. (2002). Reproduction numbers and sub-threshold endemic equilibria for compartmental models of disease transmission. *Mathematical Biosciences*, *180*(1-2), 29–48.
